# Microglia and immune signaling as architects of the brain

**DOI:** 10.3389/fncel.2026.1936860

**Published:** 2026-10-07

**Authors:** Anna Kim, Cynthia M. Solek, Edward S. Ruthazer

**Affiliations:** Montreal Neurological Institute-Hospital, McGill University, Montreal, QC, Canada

**Keywords:** astrocytes, complement, cytokines, neurodevelopment, microglia, neuroimmune, neurogenesis, synaptic pruning

## Abstract

The focus of studies on immune signaling in the CNS is often placed on the effects of inflammation and overactive immune mechanisms in the context of neurodevelopmental and neurodegenerative pathology. However, many immune regulatory pathways are also essential for healthy neuronal development and proper circuit refinement. Appropriate levels of cytokines and chemokines along with many other immune signaling and phagocytic pathways are required for normal neurodevelopment. The complement pathway and other “eat me” signals often studied in the context of inflammation and neurodevelopmental disorders, likewise play a crucial role in this process. Immune receptors and membrane-associated molecules regulate growth, differentiation and the refinement process to help establish a functional network. This review will focus on the roles that immune signaling mechanisms play in neurodevelopment of healthy animals.

## Introduction

As the primary resident immune cells of the central nervous system (CNS), microglia perform a multitude of tasks both during development and throughout life in all vertebrate animals. Many of these vital contributions to developmental and homeostatic programs are highly conserved across species, with some notable differences observed in primates (Geirsdottir et al., 2019). In addition to protecting the nervous system against various sources of infection (Eyo and Wu, 2019), microglia secrete neurotrophic factors, participate in synaptic pruning and plasticity, and remove dead and apoptotic cells (Whitelaw et al., 2022). Moreover, dysregulation of microglia is associated with neurodevelopmental and neurodegenerative disorders (Mastenbroek et al., 2024; Solek et al., 2018; Smail and Lenz, 2024; Gao et al., 2023; Nakaso, 2024).

Microglia were first described as the “third glial cell” in 1919 by Spanish neuroscientist Pio del Rio-Hortega, which he proposed to be primarily mesoderm-derived scavengers of cellular debris [(Rio-Hortega, 1919a,b,c,d), see (Sierra et al., 2016) for translation]. For many decades thereafter, microglia were regarded as dormant or resting and ramified in the absence of immunological challenge (Barron, 1995). Upon injury or infection, they were thought to become “activated,” adopting a more amoeboid shape, increasing their motility and expressing a set of marker antigens also found in circulating monocytes. However, the advent of intravital imaging technology allowed a first observation of these cells in live animals and the demonstration that microglia are highly dynamic in their “resting” state (Nimmerjahn et al., 2005; Davalos et al., 2005). This led to an increased interest in the role of microglia in normal development and the adoption by many researchers of the more apt term “homeostatic” microglia. The following years of research established microglia as key players in successful brain development and wiring, with characterization of roles in neurogenesis, developmental neuronal apoptosis, and synaptic pruning (Paolicelli and Ferretti, 2017). In addition, findings from genome-wide association studies (GWAS) revealed that many immune genes expressed by microglia are implicated in psychiatric, neurodevelopmental (Network, 2015; Voineagu and Eapen, 2013), and neurodegenerative disorders (Holtman et al., 2024). This further spurred interest in a better characterization of microglial contributions to brain homeostasis and how these processes are dysregulated in various pathologies.

While microglia contribute the bulk of immune surveillance and signaling in the developing brain, other immune cells also play a role in CNS formation and refinement. For example, maternally-derived microchimeric microglia are found in the progeny's brain and help colonization of the brain by fetal microglia (Schepanski et al., 2025). The presence of these cells helps establish a stable microglial population and has a role in regulating synaptic pruning. Yolk sac-derived border associated macrophages are also present and populate the brain during development (Utz et al., 2020). Adaptive immune cells, such as B-1a lymphocytes and CD4+ T cells promote oligodendrogenesis and microglial maturation respectively (Pasciuto et al., 2020; Tanabe and Yamashita, 2018).

In this review, we will focus on the signaling molecules that are produced by microglia and those that recruit microglia to the sites of their activity. We will give an overview of the role of cytokines and chemokines, the complement system, membrane associated molecules and reactive oxygen species (ROS) in microglial function in developmental programs, including neurogenesis, synapse formation, maturation, and pruning.

## Cytokines and chemokines

Cytokines are a broad category of small proteins used primarily by the immune system for cell signaling. Chemokines are a specialized set of cytokines that can induce a chemotactic response in cells expressing the corresponding receptors. Microglia secrete and respond to an array of cytokines and chemokines, many of which participate in neurodevelopment (Figure 1). One prominent class of these signaling molecules is pro-inflammatory cytokines, whose primary function is to respond to injury and infection by activating defense and repair mechanisms (Kany et al., 2019; O'Shea and Murray, 2008). Their functions have been extensively characterized in animal models of disease, and additional correlative evidence has been provided from human samples. In addition, several different avenues to assess cytokine function in human microglia are being pursued, including brain organoids (Yu et al., 2025), xenotransplantation of human microglia into mouse brain organoids (Popova et al., 2021), single-cell RNA sequencing (scRNA-seq) of isolated human fetal microglia (Kracht et al., 2020) as well as embryonic stem cells (ESCs), and induced pluripotent stem cell (iPSC)-derived microglia in culture or co-culture (Schepanski et al., 2025; Han C. Z. et al., 2023; Mordelt et al., 2026).

**Figure 1:**
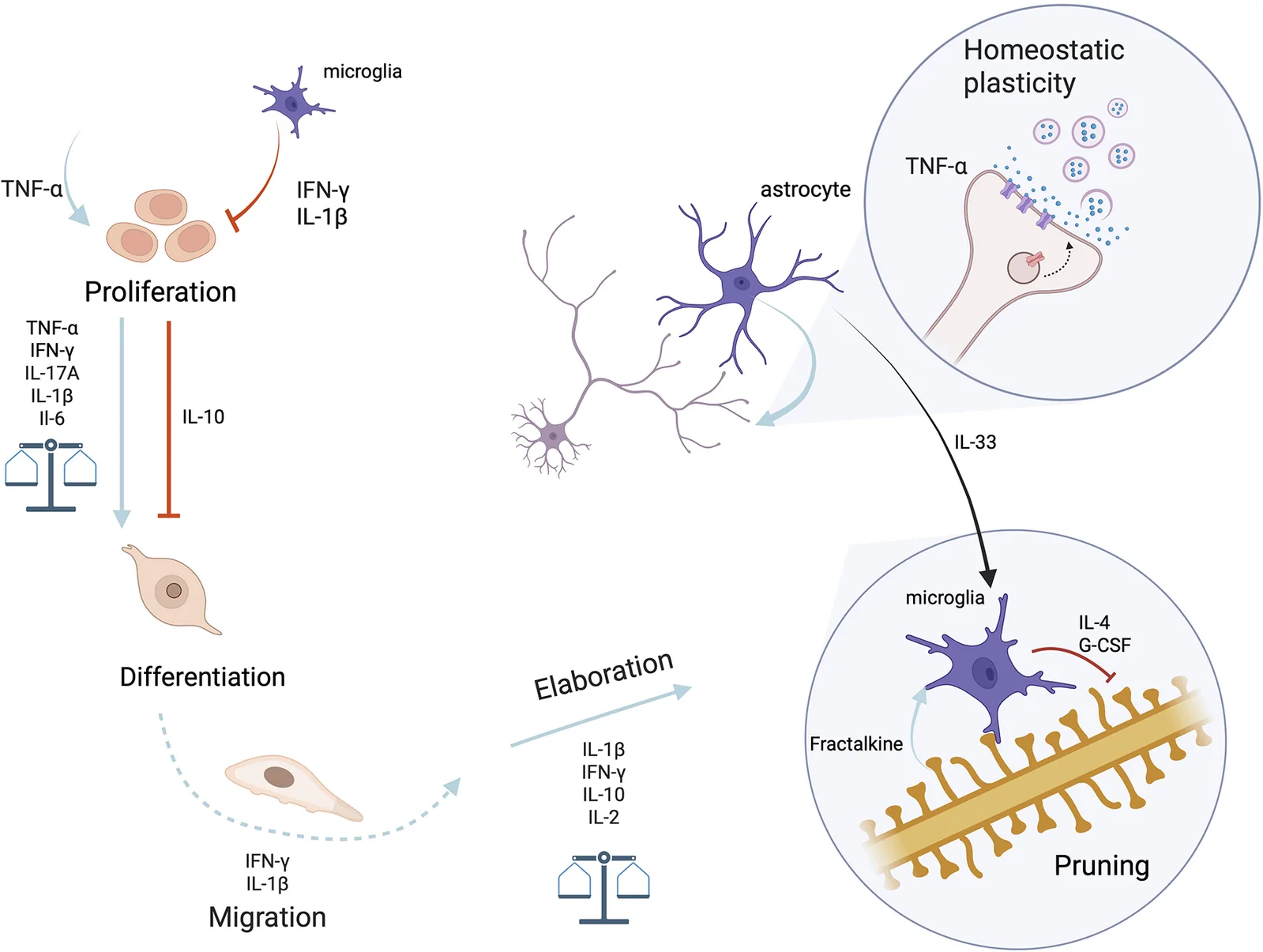
Cytokines and chemokines regulate multiple stages of neurodevelopment, from NPC proliferation to synaptic refinement. TNF-α promotes expansion of the neural precursor cell pool, whereas IFN-γ and IL-1β inhibit proliferation. Optimal levels of TNF-α, IFN-γ, IL-17A, IL-1β, and IL-6 support the differentiation of NPCs into committed postmitotic cells, whereas IL-10 delays differentiation. During neuronal migration, IFN-γ and IL-1β guide immature neurons toward their appropriate locations. Following migration, regulated levels of IL-1β, IFN-γ, IL-10, and IL-2 promote axonal growth and synapse formation to establish neural connectivity. At mature synapses, astrocyte-derived TNF-α mediates homeostatic synaptic plasticity by increasing postsynaptic AMPA receptor expression. Concurrently, astrocytic IL-33 and neuronal FKN recruit microglia to refine synaptic connections through pruning, a process that can be inhibited by IL-4 and G-CSF. Created in BioRender. Ruthazer, E. (2026) https://BioRender.com/ks90kn9.

### Fractalkine

Fractalkine (FKN, also known as C-X_3_-C motif chemokine ligand 1-CX3CL1) is a membrane-bound protein that mainly mediates cellular adhesion, but which can be cleaved by proteases to produce its soluble form (sFKN) (Gunner et al., 2019). Both the membrane bound and soluble forms of FKN bind exclusively to C-X_3_-C motif chemokine receptor 1 (CX3CR1) and modulate microglial activity via downstream signaling cascades (Harrison et al., 1998; Tarozzo et al., 2002, 2003; Maciejewski-Lenoir et al., 1999). FKN signaling is important for the regulation of neuroinflammation and neurological disease progression, synaptic plasticity, remyelination and maintaining neural homeostasis (Chen Y. et al., 2026). In physiological settings, neurons secrete FKN to attract microglia and the interaction of membrane bound FKN with CX3CR1 is thought to maintain brain homeostasis and ensure neuronal viability (De Felice et al., 2022; Goshi et al., 2020; Wood and Singer, 2025).

Correlative evidence of FKN roles in brain development comes from human studies and animal models of neurodevelopmental disorders (NDDs). A meta-analysis of gene expression profiles showed a significant down-regulation of CX3CR1 expression in schizophrenia patients compared to healthy controls (Bergon et al., 2015). FKN levels were also shown to be significantly lower in an autism spectrum disorder (ASD) patient group compared to control subjects (Kirsan et al., 2025). An analysis of human cohorts showed a genetic association of the Ala55Thr variant of CX3CR1 with ASD and schizophrenia phenotypes (Ishizuka et al., 2017).

In maternal immune activation (MIA) models in rodents, polyinosinic:polycytidilic acid [poly(I:C)] caused a decrease in microglial expression of CX3CR1 along with working memory and social behavior impairments (Mattei et al., 2017). Bacterial lipopolysaccharide (LPS)-induced maternal immune activation likewise showed a decrease in microglial CX3CR1 protein expression and a morphological phenotype in the hippocampus and dentate gyrus of male mice (de Cossio Fernandez et al., 2021).

Disruptions of FKN signaling suggest crucial roles in brain development. Indeed, in CX3CR1 knockout (KO) mice, microglial colonization of the hippocampus and somatosensory cortex in the postnatal brain is reduced compared to heterozygous or wildtype (WT) littermates (Paolicelli et al., 2014; Hoshiko et al., 2012). A greater number of apoptotic neurons was detected in the motor cortex of CX3CR1 KO mice (Ueno et al., 2013), suggesting a role for FKN signaling in the regulation of neuron number during normal development. CX3CR1 KO rats exhibit decreased hippocampal neurogenesis compared to WT animals, and interference with FKN signaling with CX3CR1 antibodies led to a decrease in the survival of immature neurons in young rats (Bachstetter et al., 2011). In FKN KO mice, cognitive impairments in long-term memory and spatial learning and memory were correlated with deficits in both hippocampal neurogenesis and long-term potentiation (LTP), which can be rescued by exogenous introduction of sFKN (Winter et al., 2020).

CX3CR1 signaling has also been shown to be important for synaptic pruning, the removal of excess or unused synapses during neural circuit refinement (Faust et al., 2021). In the mouse, significantly more PSD95 puncta were found in CX3CR1 KO hippocampal neurons, with a concurrent increase in dendritic spines and number of immature synapses at postnatal day 15 (P15) compared to WT littermates (Paolicelli et al., 2011). Downregulation of CX3CR1 in anterior cingulate cortex at P21 showed increased spine density and miniature excitatory post-synaptic current (mEPSC) frequency, suggestive of an impairment in pruning (Cao et al., 2021).

CX3CR1 KO mice also revealed a crucial role for FKN signaling in regulating the maturation of excitatory synapses during normal development. Hoshiko and colleagues found a delay in the maturation of N-methyl-D-aspartate (NMDA) receptor current kinetics at thalamocortical synapses during the second postnatal week in CX3CR1 KO mice compared with WT littermates, as well as reduced α-amino-3-hydroxy-5-methyl-4-isoxazolepropionic acid (AMPA) receptor content (Hoshiko et al., 2012). Conversely, exogenous application of FKN to hippocampal slices from 1 month old mice resulted in potentiation of NMDAR function due to the induction of D-serine release from microglia (Scianni et al., 2013). A study by Zhan et al. (2014) using whole-cell recording of Schaffer collateral synapses onto hippocampal CA1 pyramidal neurons, found that the ratio of spontaneous to miniature excitatory postsynaptic currents (EPSCs) was chronically reduced in the CX3CR1 KO mice at P38-P40, suggesting a failure to develop mature multisynapse boutons and therefore less efficient postsynaptic transmission when microglial function is compromised. CX3CR1 KO adult mice (10–12 weeks) were also shown to have impaired long-term potentiation in the hippocampus along with alterations in behavior, including a more anxious phenotype and memory encoding deficits (Mendez-Salcido et al., 2022; Rogers et al., 2011).

Hence, FKN has many demonstrated roles in brain development, from regulation of neuronal survival, to synaptic pruning, to modulation of synapse maturation. This highlights the crucial role of microglial signaling in healthy neurodevelopmental programs.

### Interleukin-17A

Interleukin-17A (IL-17A) has long been viewed primarily as a key mediator of the neuroinflammatory pathways that cause or worsen neurodegenerative diseases (Chen et al., 2020). However, some roles of IL-17A in developmental programs are beginning to emerge. The expression levels of this cytokine are very low in the developing brain; however the IL-17A receptor is highly expressed in microglia (Das Sarma et al., 2009). Given that it shares protein structure elements with nerve growth factor (Hymowitz et al., 2001), it has been postulated to have neurotrophic properties. Studies examining serum levels of cytokines in human children have established a link between elevated IL-17A expression and ASD (Suzuki et al., 2011; Al-Ayadhi and Mostafa, 2012; Jones et al., 2017; Sallam et al., 2024; Barbosa et al., 2020; Hill et al., 2025), although one study reports a significant decrease in serum IL-17A levels in cases compared to cohort controls (Casey et al., 2022). Its levels are also reported to be reduced in children born following maternal Zika virus infection who exhibit microcephaly and intellectual disability (Fialho et al., 2023). This suggests that careful regulation of this cytokine is required for proper neuronal development. Indeed, a study using human iPSC-derived neural progenitor cells (NPCs) showed that application of IL-17A in culture increased expression of synaptic and neuronal polarity proteins in these cells (Gomes et al., 2022). In rodent model experiments, expression of synaptophysin and PSD95 proteins appeared to be decreased in neonatal (P6) IL-17A KO mice (Zhang Q. et al., 2025), further supporting a role for this cytokine in neurodevelopment. Increase in IL-17A levels during gestation, through prenatal stress (Amani et al., 2021), SARS-CoV-2 spike protein exposure (Erdogan et al., 2023), intraperitoneal injections of the viral mimic poly(I:C) (Garay et al., 2013) or of LPS (Erdogan et al., 2023; Yasumatsu et al., 2020), gestational hypothyroxinemia (Gonzalez-Madrid et al., 2024), maternal overexpression (Murakami et al., 2021) or recombinant IL-17 protein injection (Choi et al., 2016; Sasaki et al., 2020; Gumusoglu et al., 2020) were shown to correlate with a combination of changes in microglial morphology, cortical anomalies and/or rodent behavioral changes. Conversely, a decrease of IL-17A levels was detected in the hippocampus of P60 mice with autistic-like behavior following MIA (Dutra et al., 2023). Moreover, IL-17A helps to increase the inflammatory response by promoting the production of other cytokines, including IL-1β, TNF-α, and IL-6 (Choi et al., 2016), which provides further evidence for a potential role for this immunomodulator in neurodevelopmental programs. The precise mechanisms that are regulated by IL-17A during normal development remain to be fully characterized.

### Interleukin-6

Interleukin-6 (IL-6) was first identified as a maturation factor for B-cells (Hirano et al., 1985), however, pleiotropic functions for this cytokine were rapidly characterized including on other immune cells, blood vessels, hepatocytes and neuronal cells (Kishimoto et al., 1995). Characterization of the effects of IL-6 on neuronal development are complicated by the intricate network of signaling molecules and receptors which converge on common intracellular pathways (Heinrich et al., 2003) and the multiple sources of IL-6 expression in the developing brain (Cornfield and Sills, 1991; Schobitz et al., 1993; Yan et al., 1992; Gadient and Otten, 1993, 1994a,b; Vallieres and Rivest, 1997).

Microglia express IL-6 in many inflammatory circumstances (Bobbo et al., 2019) but also express the IL-6 receptor-alpha chain (IL-6R), which binds IL-6, and the trans-membrane signaling subunit, glycoprotein 130 (gp130) (Burton et al., 2013; Couch et al., 2023). This places them in a position to mediate many of the different functions that IL-6 performs during neuronal development. Furthermore, maternally derived IL-6 was shown to influence embryonic microglial morphology in a mouse model of prenatal stress (Gumusoglu et al., 2020). In a murine MIA model, Pratt et al. (2013) showed that microglia at embryonic day 16.5 (E16.5) adopt an activated, inflammatory phenotype in maternal poly(I:C) injected WT but not IL-6 KO mice. Interestingly, CD11b-negative (non-microglial) fractions from IL-6 KO fetal mice, nonetheless, exhibited high basal expression of inflammatory cytokines even in saline-injected controls. This further supports the assertion that precisely controlled levels of IL-6 are necessary in the CNS for healthy development.

The specific roles of IL-6 in neurodevelopment are somewhat controversial but include regulation of neural stem cells (NSCs) both *in vitro* (Niwa et al., 1998) and *in vivo* (Bowen et al., 2011; Gallagher et al., 2013). IL-6 and its soluble receptor, sIL-6R, were shown to induce early neurogenesis and to initiate neuronal differentiation of human NSCs (Sulistio et al., 2018), and to promote neuronal differentiation in rat pheochromocytoma (PC12) cells in culture (Sterneck et al., 1996; Marz et al., 1997). On the other hand, IL-6 significantly reduces adult mouse hippocampal neuronal differentiation via the JAK/STAT3 pathway (Kong et al., 2019). IL-6 also has a potential neurotrophic role, as it was shown to promote the survival of postnatal rat neurons in culture (Hama et al., 1989, 1991). Moreover, inflammatory cytokines, including IL-6, can act as neuromodulators and influence neuronal excitability, in part by signaling through astrocytes (Vezzani and Viviani, 2015). Finally, increased expression of IL-6 either directly or through an infection model has been shown to cause abnormalities in neuroanatomical structural development and to impair neuronal plasticity [reviewed in (Wei et al., 2013)]. As one of the core pro-inflammatory cytokines expressed by microglia during development, IL-6 is a signaling factor that can influence multiple facets of neurodevelopment and neuromodulation.

### Interleukin-1β

Interleukin-1β (IL-1β) is produced in the CNS by microglia (Giulian et al., 1986). As with most cytokines, the roles of IL-1β in pathology are much better characterized than its functions in healthy development. IL-1β is one of the main immune modulators linked to neuronal degeneration in Alzheimer's disease (Griffin and Mrak, 2002) and is at the center of signaling pathways for many additional cytokines (Kaneko et al., 2019). During neurodevelopment, elevated levels of IL-1β have been correlated with deficits in various rodent models of NDDs, from inflammatory activation of microglia to altered behavior indicative of pathology [reviewed in (Solek et al., 2018; Szabo et al., 2025)].

IL-1β is expressed at high levels in the brain during development, suggesting an essential role in neuronal development (Ma et al., 2014; Mousa et al., 1999; Giulian et al., 1988). *In vitro* studies showed that IL-1β influences the migration of embryonic (E14) mouse astrocyte progenitor cells in neurospheres by modulating calcium signaling (Striedinger and Scemes, 2008) and of juvenile (P15) rat cortical neurons in a dose-dependent manner (Ma et al., 2014). Further *in vitro* evidence suggests that IL-1β signaling contributes to the decrease in proliferation of E14 rat NPCs isolated from ventral mesencephalon (Crampton et al., 2012) and to their differentiation, likely through the Wnt5a/RhoA/JNK pathway (Park et al., 2018). IL-1β also has the potential to influence axonal growth cone turning of migrating neurons and is involved in the regulation of neurite outgrowth, as it acts synergistically with neurotrophin-3 to stimulate neurite density and length (Boato et al., 2011). *In vivo* inhibition of IL-1R1 in rat cortical neurons by RNAi resulted in a decrease in radial migration of these neurons at P5 (Ma et al., 2014), further supporting a role for IL-1β signaling in neuronal migration. Finally, IL-1β levels appear to need tight regulation in zebrafish axon arborization in larval stages, since both elevated levels and inhibition of expression of this cytokine led to alterations in retinal ganglion cell branching (Solek et al., 2021).

An instance of gene polymorphism in the IL-1β gene has been identified and is associated with increased incidence of ASD in humans (Pekkoc Uyanik et al., 2022), although it is unclear whether the genetic variants result in either gain- or loss-of-function phenotypes. Together, these data point to important roles for IL-1β in both healthy neuronal development and in pathological consequences resulting from either increased or decreased levels of expression.

### Tumor necrosis factor alpha

Tumor necrosis factor alpha (TNF-α) is another pro-inflammatory cytokine that has been implicated in neuronal development and pathology. It is often upregulated alongside IL-1β and IL-6 in inflammatory environments (Solek et al., 2018; Zhang et al., 2026) with some exceptions (Chen Q. et al., 2026). As with many other signaling molecules involved in the CNS, the effects of TNF-α are not straightforward. For instance, high concentrations of TNF-α treatment have been shown to induce apoptosis of cultured neonatal mouse NPCs, while low concentrations of this cytokine promoted NPC proliferation and differentiation (Bernardino et al., 2008), suggesting a complex role for TNF-α in postnatal neurogenesis. Several roles for TNF-α secreted by microglia in healthy brain development have been postulated, although many are likely to be indirect. For instance, CXCR4 signaling in the presence of TNF-α was shown to increase intracellular Ca^2+^ levels and glutamate release in rat and human ESC-derived astrocytic cells in culture (Bezzi et al., 2001). Astrocytes have well-characterized and evolutionarily ancient roles in neuronal development, including in neuronal migration, axon specification and growth, neuronal differentiation and establishment and maintenance of proper synaptic connectivity (Allen and Eroglu, 2017; Chung et al., 2015; Allen and Lyons, 2018). TNF-α signaling acts as a permissive factor for the glutamate release step of astrocytic gliotransmission in the mouse dentate gyrus (Santello et al., 2011). Moreover, TNF-α can modulate excitability of all classes of voltage-gated channels, most notably voltage-gated sodium channels, a phenomenon extensively documented for peripheral dorsal root ganglion neurons in the context of neuropathic pain (Vezzani and Viviani, 2015).

TNF-α can also modulate the composition of GABA and glutamate receptor subunits at neuronal membranes; the cytokine-induced changes resulting in increased neuronal network excitability (Vezzani and Viviani, 2015). Interestingly, TNF-α released by astrocytes is responsible for the homeostatic “up-scaling” of AMPAR-mediated synaptic transmission in cortical neurons in response to chronic neural activity blockade and to monocular deprivation during the developmental critical period for ocular dominance plasticity (Kaneko et al., 2008; Heir et al., 2024). In the developing Xenopus tadpole visual system, chronic exposure to TNF-α accelerated the developmental unsilencing of immature NMDAR-only synapses via trafficking of AMPARs to retinotectal synapses, and led to expanded dendritic arborizations of optic tectal neurons (Lee et al., 2010). Therefore, TNF-α is poised to be a critical regulator of neuronal development, with closely controlled levels produced by both microglia and astrocytes being necessary for healthy circuit wiring and function.

### Interleukin-33

Interleukin-33 (IL-33) is mainly secreted by astrocytes in development, and its expression decreases in the mature brain (Wicher et al., 2013), although it is also expressed by oligodendrocytes (Allan et al., 2016) and hippocampal neurons (Nguyen et al., 2020). This crucial cytokine, which functions as an alarmin and helps promote the immune response following cellular necrosis (Gadani et al., 2015), has demonstrated roles in neurodevelopment [reviewed in (Luo and Yang, 2026)]. It was shown to influence microglial synapse engulfment in the developing mouse thalamus and spinal cord, as animals with IL-33 conditionally deleted in astrocytes showed increased excitatory and inhibitory synapse numbers at P30 and fewer PSD95 puncta engulfed by microglia at P15 (Vainchtein et al., 2018). Conversely, exogenous introduction of excess IL-33 in the developing mouse brain at P15 resulted in reduced number of VGLUT-1 synapses in somatosensory thalamus and increased microglial engulfment of synaptic material (Han R. T. et al., 2023). Loss of neuronal expression of IL-33 or deletion of *Il1rl1* (ST2), the receptor for IL-33 in microglia, resulted in impaired spine plasticity, reduced newborn neuron integration, and memory task deficits (Nguyen et al., 2020). IL-33 has also been shown to regulate microglial phagocytic activity in mouse cortical development in a process that involves mitochondrial regulation of bioenergetic metabolism in microglia, since disruption of the ST2-AKT pathway resulted in a reduction of IL-33 induced synaptic engulfment by microglial BV2 cells *in vitro* (He et al., 2022). Finally, IL-33 is involved in oligodendrocyte precursor cell maturation and myelination since both processes were impacted in IL-33 KO mice (Sung et al., 2019). Overall, signaling of IL-33 through microglia has many demonstrated roles in brain development but also in continued brain health.

### Interferon-γ

Interferon-γ (IFN-γ) is another pro-inflammatory cytokine produced by microglia. Elevated gestational and perinatal levels of IFN-γ have been found in some mothers of children with NDDs (Jones et al., 2017; Allswede et al., 2016), and in the patients themselves (Masi et al., 2015), suggesting that dysregulation of this cytokine may contribute to neurodevelopmental abnormalities. IFN-γ has been shown to negatively regulate the neuronal stem cell niche in mice *in vivo*, and *in vitro* neurosphere formation from NPCs isolated from P2 and adult mice is reduced in the presence of excess IFN-γ (Li L. et al., 2010). This effect curiously was greater in the absence of microglia, suggesting they may release factors that protect against IFN-γ-mediated inhibition. IFN-γ was also shown to promote neuronal differentiation and neurite outgrowth in rat, mouse and human NSCs *in vitro* (Wong et al., 2004; Johansson et al., 2008; Leipzig et al., 2010; Song et al., 2005), and acts through microglia to enhance neurogenesis in mouse NPC co-cultures (Butovsky et al., 2006). Moreover, IFN-γ also promotes migration and reduces proliferation and cell survival of neural progenitors (Wong et al., 2004; Song et al., 2005; Ben-Hur et al., 2003). Conversely, IFN-γ was shown to inhibit neuronal differentiation of mouse NPCs *in vitro* and in the E14.5 mouse brain *in vivo* (Ahn et al., 2015). IFN-γ was also shown to confer neuroprotection against apoptosis via an Erk1/2-dependent mechanism (O'Donnell et al., 2015). Finally, studies in IFN-γ KO mice revealed behavioral impairments and defects in neuronal connectivity (Filiano et al., 2016), suggesting that some level of expression of this cytokine is necessary for proper neuronal development and that alterations in those levels are likely to have functional consequences.

### Other cytokines and chemokines

Many other cytokines and chemokines have potential roles in neurodevelopment although their functions have mostly been investigated in the context of neuroinflammation [reviewed in (Zhang et al., 2026)]. A few studies have hinted at developmental functions for these signaling molecules in development. For instance, increased levels of Granulocyte Colony-Stimulating Factor (G-CSF) in mice led to increased dendritic spine density, a higher proportion of immature spines in the medial prefrontal cortex and altered social behavior in the offspring (Kirikae et al., 2026). The anti-inflammatory cytokine IL-10 is mostly investigated in the context where it mitigates the negative effects of neuroinflammation (Lobo-Silva et al., 2016). However, it has also been shown to regulate neurogenesis by delaying rat and mouse neural progenitor differentiation *in vitro* (Perez-Asensio et al., 2013), to promote neurite outgrowth in juvenile (P16-18) rat primary cortical neurons in culture (Chen et al., 2016) and to be important for microglia-induced synapse formation in cultured embryonic (E18) rat hippocampal neurons (Lim et al., 2013). IL-4, another anti-inflammatory cytokine, has been implicated in learning and memory, as IL-4 KO mice exhibited cognitive deficits which could be rescued by transferring WT T-cells (Derecki et al., 2010). Prematurely high levels of IL-4 in the mouse brain caused reduced microglia-mediated pruning of whole granule cells (GCs), excessive connectivity from parallel fibers onto Purkinje cells (PCs), and an attention-deficit/hyperactivity disorder (ADHD)-like phenotype (Guedes et al., 2023). IL-2 has long been known to have effects on neurons including promoting cortical, hippocampal and striatal neuron survival, extension of neurites and the activation of microglia (Hanisch and Quirion, 1995), although more detailed characterization of its contribution in neurodevelopment has been minimal (Petitto et al., 2012). And even though it was shown to be negatively correlated with ASD in GWAS datasets (Guedes et al., 2023; Liu et al., 2025), Il-2 is infrequently reported in human and animal model studies of NDDs. In addition, many chemokines besides FKN signal through microglia and have demonstrated roles not only in brain development but also in neuropathology [reviewed in (Watson et al., 2020)].

The cytokine and chemokine landscape in neurodevelopmental processes is vast and complex (Figure 1). Besides these signaling molecules having multiple cellular targets, many of them influence the expression of an array of other cytokines and chemokines, creating a network of inter-cellular interactions which can be tricky to untangle experimentally. Disease models and animal KOs offer a window into these processes, however, understanding how these crucial immune messengers influence neuronal development, circuit formation and synaptic refinement in the healthy brain requires further investigation.

## Immune recognition and phagocytic signaling pathways

Among the immune-related pathways implicated in healthy brain development, innate immune receptors and other membrane associated signaling molecules have emerged as important regulators in neurodevelopment. Toll-like receptors (TLRs), Major Histocompatibility Class I (MHC-I) molecules, phosphatidylserine receptors and purinergic receptors are all required to sense pathogen presence and damage, and to launch a proper immune response to protect the host (Salvador, 2022; Qian et al., 2026; Liu et al., 2023; Eberhardt et al., 2022). However, studies in neurodevelopment and in neurodevelopmental disorders have also revealed their involvement in healthy brain development, indicating their roles to be more pleiotropic than initially thought.

### Major histocompatibility class I molecules

MHC-I molecules are generally known as the antigen presenting receptors found on all nucleated cells (Liu et al., 2023). They present intracellular peptides to be detected by patrolling immune cells. In turn, non-self proteins, such as viral antigens, are detected on the surface of infected cells and tagged for degradation. However, numerous studies have noted a function of MHC-I proteins in neuronal function through the modulation of neurogenesis, synaptogenesis, and synaptic plasticity during development (Corriveau et al., 1998; Huh et al., 2000; Nelson et al., 2013). Their role in the nervous system was first discovered in 1998, when Corriveau et al. (1998) noted a positive spatial and temporal correlation of MHC-I expression in the dorsal lateral geniculate nucleus (dLGN) and hippocampus with levels of neuronal activity. In addition, inhibition of spontaneous activity led to lowered expression of MHC-I on the cell surface. A subsequent study noted that knock-out of MHC-I led to a decreased segregation of contralateral and ipsilateral retinogeniculate projections to the dLGN and enhanced LTP in the hippocampus (Huh et al., 2000). Loss of neuronal cell surface expression of MHC-I molecules also led to a defect in long-term depression (LTD) in mouse hippocampal slices. It was subsequently found that MHC-I KO mice exhibited an increase in the size of the presynaptic boutons relative to WT littermates and a concurrent increase in mEPSC frequencies in hippocampal neurons (Goddard et al., 2007). Conversely, elevated levels of MHC-I in transgenic mouse neurons led to significant reductions in the presynaptic markers synaptophysin and GAP43 in the dentate gyrus and CA3 area and a modest decrease in the number of pyramidal neurons in the hippocampal CA1 region compared to WT mice (Wu et al., 2011). As MHC-I molecules are known for recruitment of immune response components, their correlation with activity may indicate their roles in microglial recruitment and identification of specific synapses for refinement of the circuit during development. Moreover, MHC-I expression can be regulated by cytokines (Neumann et al., 1997), further strengthening their roles as developmental modulators of neuronal circuits.

### Toll-like receptors

TLRs were named after their structural similarity to the Drosophila Toll receptors [reviewed in (Valanne et al., 2022, 2011)]. They were originally identified due to their effect on embryonic development, but also mediate immune responses in the fly. In mammals they are involved in innate immunity, sensing components of infectious pathogens, including bacterial wall and foreign DNA (Verma et al., 2026). Many studies now note that TLRs do not only function in immune defense but also participate in neurodevelopment.

Different TLR proteins are expressed in mammalian microglia, neurons, astrocytes and oligodendrocytes (Hanke and Kielian, 2011) and expression levels in neurons vary throughout development (Abarca-Merlin et al., 2024). Several TLRs have been implicated in neurogenesis (Barak et al., 2014). TLR2 KO mice exhibit a decrease in NPC proliferation in the dentate gyrus at P7 and a significant decrease in the number and length of neurites of TLR2 KO neurons in culture compared to WT (Rolls et al., 2007). TLR3 appears to have an opposite effect, with NPCs from TLR3 KO mice capable of forming a significantly higher number of neurospheres, and having more proliferating cells in the dorsal telencephalon at E13.5 than WT controls (Lathia et al., 2008). In addition, TLR3 KO mice exhibit increased neurogenesis in the hippocampus and deficits in learning and memory (Okun et al., 2010). Young TLR4 KO mice also show increased proliferation in the hippocampus, suggesting a role for this receptor as a negative regulator of neurogenesis (Connolly et al., 2020). In addition, TLR4 is a negative regulator of retinal progenitor cells (RPCs), since a deficiency in TLR4 results in increased proliferation and neuronal differentiation in the early postnatal mouse retina, whereas activation of TLR4 restricts RPC proliferation and differentiation *in vitro* (Shechter et al., 2008). TLR5 is able to enhance neural differentiation of mouse embryonic stem cells *in vitro*, and promotes differentiation of NSCs in the dentate gyrus (Seong et al., 2022).

TLR7 and TLR8 have overlapping but distinct roles in axon and dendritic morphology and synaptic organization (Hung et al., 2018). TLR8 activation induces dendritic pruning but has no obvious effect on axonal growth (Hung et al., 2018), whereas TLR7 influences arbor elaboration, since *Tlr7* knockdown leads to longer dendrites in cultured mouse cortical neurons (Liu et al., 2013). TLR4 signaling through Hevin, a synaptogenic protein secreted by astrocytes, is also involved in the process of thalamocortical synapse pruning in young (P15) mice (Ramirez et al., 2026). TLR activation in glia, particularly TLR10 in microglia (Verma and Kim, 2016) and TLR3 in astrocytes (Hung et al., 2018; Farina et al., 2005), has also been shown to promote expression of a number of cytokines and chemokines, including IL-6, TNF-α and IL-10. All these studies contribute to the better understanding of the roles of TLRs in the mammalian brain, where they regulate the proliferation and differentiation of NPCs into neurons or glial cells. They also control cell death and survival, neurite growth, synaptogenesis, and the modulation of spine density and size through communication with glial cells, including microglia.

### Purinergic receptors

P2Y12 receptors (P2Y12R) are purinergic receptors that have well-described roles in microglial function in neuroinflammation (Gomez Morillas et al., 2021). Their expression is a critical marker differentiating microglia from macrophages in the nervous system (Mildner et al., 2017) and is present throughout life, although expression levels vary over time (Mildner et al., 2017; Crain et al., 2009; Peng L. et al., 2019). They are activated by interaction with adenosine diphosphate (ADP), which causes microglial activation including extension of processes and phagocytic activity (Illes et al., 2021; Ohsawa et al., 2010). An extensive review of the many roles of P2Y12 receptors in microglia has been published previously (Lin et al., 2021), but here we highlight the main functions related to neurodevelopment.

Purinergic signaling, including via P2Y12, has been implicated in regulation of neurogenesis and brain development (Rodrigues et al., 2019). Microglial P2Y12 signaling has been shown to regulate neurogenesis and neuronal projections (Mo et al., 2019), as well as to promote proliferation of adult mouse subventricular zone cells (Suyama et al., 2012). KO of P2Y12 receptors in microglia leads to aberrant formation of cortical layers, suggesting a role for microglia in neuronal migration (Cserép et al., 2022). P2Y12 signaling is additionally involved in activity-dependent remodeling of neuronal networks, as microglia rapidly respond to experience-dependent changes in neuronal activity, such as monocular deprivation, and that response is abrogated in the absence of P2Y12R activity (Sipe et al., 2016).

P2Y12Rs also contribute to the microglial response to synaptic activity (Abbracchio et al., 2009). Microglia directly interact with the cell bodies of pyramidal neurons through P2Y12R-rich contacts called somatic microglial junctions (Cserép et al., 2020; Posfai et al., 2026). These junctions form and re-form in a dynamic way during development and have been detected in the ventricular zone/subventricular zone of E15 mice (Cserép et al., 2022). The loss of P2Y12R receptors in microglia leads to increased excitability of neurons in the hippocampus and increased anxiety-like behavior in mice (Peng L. et al., 2019). Both microglial depletion and genetic deletion of P2Y12R lead to increased baseline and evoked firing rates in narrow-spiking interneuron populations in the mouse barrel cortex (Kiraly et al., 2026). Moreover, mutations in the locus encoding P2Y12 receptors are observed in some epileptic patients (Wang et al., 2022), suggesting a neurodevelopmental role for these receptors in humans (Kiraly et al., 2026). Taken together, the current data point to a conserved role of P2Y12 receptors in microglial mobilization for neuronal survival, migration and refinement, where deficits lead to aberrant states caused by miswired circuits.

### Phosphatidylserine

Phosphatidylserine (PS) is a type of anionic phospholipid that is normally constrained in healthy cells to the intracellular leaflet of the plasma membrane bilayer through the activity of specific lipid transporter enzymes called “flippases” (Peet et al., 2020). It can be “flopped” to the outer membrane of the cell during cellular injury or under conditions where flippase activity is reduced (e.g., in apoptosis). In addition, PS can be actively externalized by “floppases” and bidirectional “scramblases” such as Xk-related protein 8 (Xkr8) (Neniskyte et al., 2023). Exposed extracellular PS serves as a canonical “eat me” cue that engages engulfment or pruning by phagocytic cells, such as microglia, which express diverse PS receptors (Figure 2). Furthermore, because of the high energetic demands of flippase activity, ATP depletion resulting from reduction or impairment of mitochondrial activity can also result in externalization of PS and increase phagocytic pruning (Shacham-Silverberg et al., 2018). Therefore, failure of proper PS metabolism and maintenance has been associated with a broad range of neurodevelopmental disorders, including ASD (Ma et al., 2022; Li et al., 2024).

**Figure 2:**
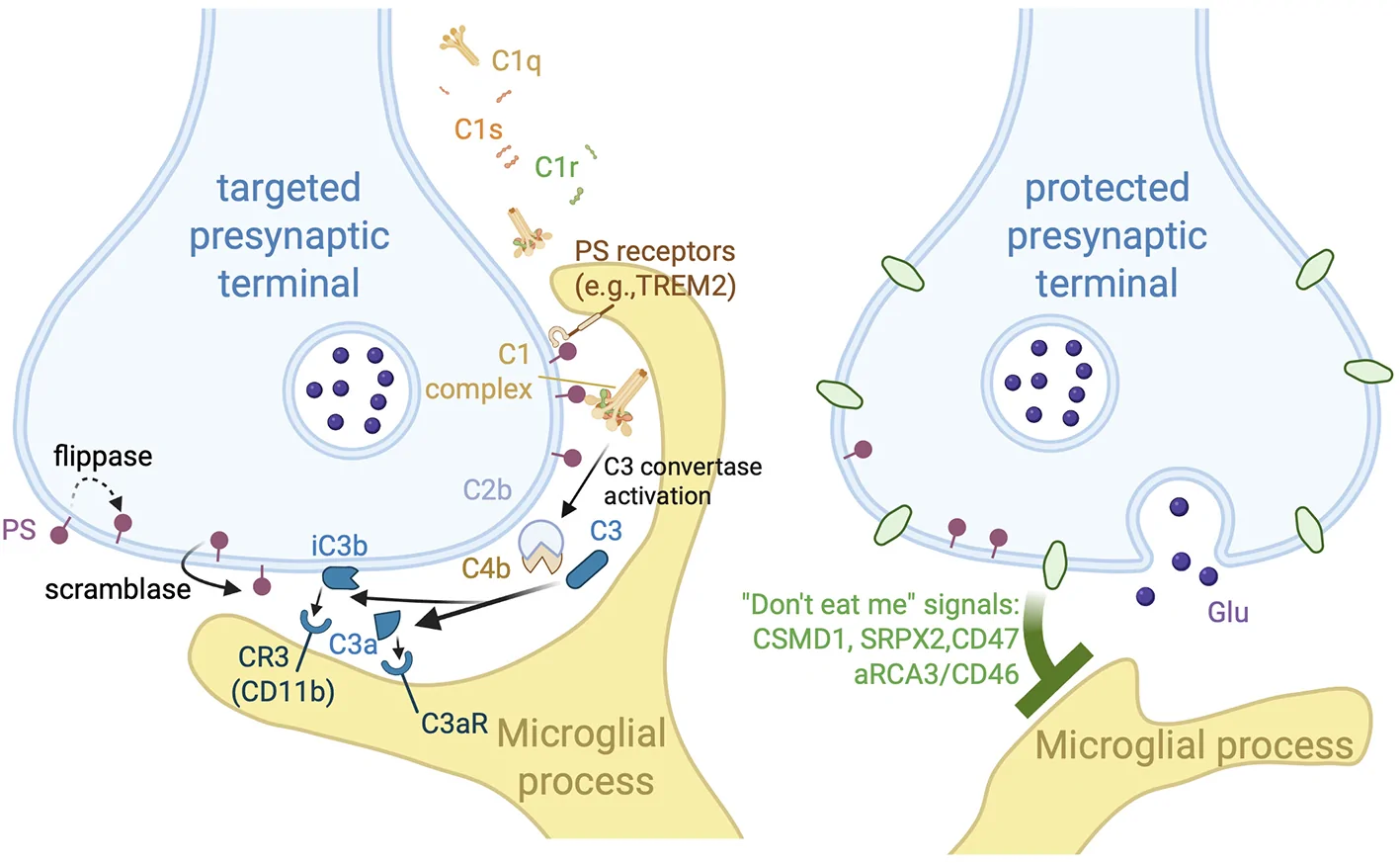
Role of the complement system in neurodevelopment. Complement system components are involved in synaptic pruning. PS exposed on the outer membrane at a synaptic terminal leads to recruitment of microglia through TREM2 signaling and C1q tagging. The C1 complex (C1qr_2_s_2_) binds to targets, leading to C1r and C1s protease activation. These cleave C2 and C4 into C2b and C4b, which comprise the C3 convertase that cleaves C3 into C3a and the C3b opsonin. C3a and C3b signal via C3aR and CR3 (CD11b) respectively on microglia to promote the engulfment of tagged synapses. Conversely, synapses can be protected from microglial pruning via “don't eat me” signals, including complement regulatory proteins such as aRCA3 (CD46), CSMD1, SRPX2 and CD47. Created in BioRender. Ruthazer, E. (2026) https://BioRender.com/zvmor78.

One example of a flippase implicated in NDD is the ATPase Phospholipid Transporting 8A1 (ATP8A1), a member of the P4-ATPase family. Elevated expression levels of ATP8A1 have been reported in adolescent ASD patients (Kerr et al., 2016). The gene encoding ATP8A1 is located in band 4p13, a locus associated with brain development and GABAergic synaptic function, and which is genetically linked to ASD and other NDDs (Polan et al., 2014). As ATP8A1 removes PS from the outer leaflet, it could potentially prevent PS signaling for synaptic pruning by glia (Raiders et al., 2021). Indeed, lentiviral overexpression of ATP8A1 in hippocampus does result in a reduction in synapse number and strength, however ATP8A1 KO mice appear to exhibit a similar phenotype (Kerr et al., 2016).

Conversely, flippase loss-of-function can result in excessive pruning of neurons due to increased exposure of PS in the outer membrane leaflet. CDC50A is a chaperone protein for phospholipid flippases such as ATP11A (Li et al., 2021). In the developing nervous system, its expression is positively regulated by neural activity, where it limits PS exposure at the cell surface. Knockdown of CDC50A results in increased engulfment of presynaptic axon terminals by microglia and induces synapse elimination in the retinogeniculate projection, consistent with PS acting to promote synaptic phagocytosis in an activity regulated manner. Interestingly, postsynaptic KO of CDC50A was found to selectively promote elimination of inhibitory synapses, an effect that was prevented in mice in which the microglial phagocytic receptor MERTK, a member of the TAM (Tyro-3, Axl, Mertk) family of receptor tyrosine kinases, was knocked out (Park et al., 2021).

Another well-characterized microglial PS receptor is the S4 splice variant of the adhesion G protein-coupled receptor GPR56, a PS-binding protein that is expressed at high levels in microglia during development (Li et al., 2020). GPR56 KO mice exhibit reduced microglial engulfment of PS, along with increased synapse densities in the dLGN and hippocampus. Furthermore, segregation of RGC inputs in the dLGN at P10 is impaired in GPR56 KO mice.

Initially identified through its association with neurodegenerative disorders, including Alzheimer's (Guerreiro and Hardy, 2013) and Parkinson's (Colonna and Butovsky, 2017) diseases, Triggering Receptor Expressed on Myeloid cells 2 (TREM2) is another PS receptor that contributes to developmental synapse elimination by microglia (Filipello et al., 2018; Scott-Hewitt et al., 2020). TREM2 KO mice reveal higher densities of excitatory synaptic markers, as well as increased mEPSC frequency, in the hippocampus at P18-20. WT hippocampal neurons co-cultured with microglia for 24 h results in a microglia-mediated reduction in synapse number. However, this reduction was abrogated when microglia from TREM2-lacking mice were used. In a mouse model of childhood adversity, it was found that deprivation by limited bedding reduced TREM2 levels which contributed to impaired microglial pruning in the hippocampus (Ahmed et al., 2026).

Other PS receptors may be required for PS recognition and participate in phagosome formation and stabilization. A study of microglial neuronal engulfment in embryonic zebrafish noted that the PS receptors BAI-1 and TIM-4, actively participate in the mechanics of phagocytosis (Mazaheri et al., 2014). Morpholino knockdown of BAI-1 led to a doubling of the time required for the formation of phagosomes by the microglia, while the KO of TIM-4 led to the formation of unstable phagosomes, prone to losing their cargo, in turn preventing effective responses to PS displayed on neurons.

## Reactive oxygen species

Reactive oxygen is produced as a byproduct of oxidative phosphorylation, and is often regarded as a harmful, damaging radical molecule associated with pathological states. Many neurodegenerative disorders involve an increase in ROS, which in turn is associated with oxidative stress and cellular damage (Oswald et al., 2018a). ROS is often generated as a response to neuroinflammation. ROS can also activate the inflammasome, a cytoplasmic multiprotein complex that increases inflammatory response (Yang et al., 2013).

However, ROS is also an important physiological signaling molecule in neurodevelopment. NSCs are capable of maintaining high ROS levels intracellularly, which they use as a second messenger to promote proliferation over quiescence (Le Belle et al., 2011). Hippocampal neurons also have been found to express NADPH oxidases and NOX2, which generate ROS during development. Inhibition of ROS production leads to delayed growth and reduced axon lengths (Wilson et al., 2015). Concentrations of ROS appear to reflect activity levels in neurons (Bindokas et al., 1996), and studies noted that submerging hippocampal slices in superoxide scavengers can prevent NMDAR-mediated long-term potentiation by preventing appropriate modulation of Protein Kinase C activity (Klann et al., 1998). Conversely, an increase in ROS is sufficient to inhibit both evoked and spontaneous synaptic vesicle fusion and neurotransmitter release at the mouse and frog neuromuscular junctions through its impairment of SNAP25 (Giniatullin et al., 2006).

Activity-dependent synaptic refinement in the Drosophila model has also been demonstrated to depend on ROS generation. Excess excitation of motor neurons was found to promote mitochondrial metabolic ROS-activated stress signaling to alter neuromuscular junction architecture both pre- and postsynaptically (Oswald et al., 2018b; Peng J. J. et al., 2019).

## Caspase-3

Caspases are a family of cysteine-dependent, aspartate-directed proteases that are responsible for regulating programmed cell death (apoptosis) and certain developmental processes. Many apoptotic pathways converge onto caspase-3 and its activation is often recognized as the “point of no return” for cell death (Porter et al., 1999). Caspase-3 function has also been implicated in neuronal development and synaptic refinement. In the developing retinotectal system of Xenopus tadpoles, it was demonstrated that plasticity-inducing visual stimulation could drive caspase activation, leading to the rapid degradation of MEF2, which in turn altered both visual response properties and dendritic remodeling by tectal neurons (Chen et al., 2012). This result is consistent with demonstrations in mouse hippocampus that NMDAR-dependent LTD (but not LTP) requires mitochondrial activation of caspase-3 (Li Z. et al., 2010). In the developing mouse retinogeniculate projection, caspase-3 levels were found to be upregulated in postsynaptic dLGN neurons when the retinal afferents from one eye were silenced by monocular viral expression of tetanus toxin, but not when both eyes were silenced (Yu et al., 2025). In Casp3 KO mice, activity-dependent anatomical segregation of retinal afferents into eye-specific laminae in the dLGN is disrupted, along with a failure to prune the number of functional inputs onto individual dLGN neurons measured by whole-cell recording. Moreover, microglial engulfment of axon terminals is significantly reduced in the Casp3 KO mice.

In another study microglia were co-culltured with hippocampal neurons expressing hM3Dq, a designer receptor exclusively activated by designer drugs (DREADD), which increases neural activity upon addition of clozapine-N-oxide (Andoh et al., 2025). Using immunocytochemistry for cleaved caspase-3 as well as a fluorescent reporter of caspase-3 activity, they observed increased caspase-3 activation in axons and increased microglial engulfment of presynaptic material. Interestingly, decreased activity via inhibitory DREADD expression did not affect either caspase-3 activation or microglial engulfment compared to controls, indicating that caspase activity is not perfectly coupled with neuronal activity. Furthermore, the study showed by fluorescent labeling of PS and complement C1q protein that elevating caspase-3 activity promoted the targeting of these “eat me” signals to presynaptic sites. This finding supports reports that caspase-mediated inactivation of flippases may underlie PS externalization at the plasma membrane (Mandal et al., 2002).

## Complement system in neurodevelopment

The complement system consists of a series of extracellular proteins that aids in clearing pathogens and unwanted cellular debris as a part of innate immunity (Taylor et al., 1998). Beyond the well-characterized functions in innate immunity, roles for the complement system have been identified in neurodevelopment, including in neurogenesis, neuronal migration and circuit wiring refinement (Magdalon et al., 2020). Most of the protein components of the complement system are expressed locally in the brain during embryonic and early postnatal development (Zhang Y. et al., 2025). To date, complement C3, C4 and C1q have been most extensively associated with neurodevelopmental functions, with other proteins also implicated in neuronal circuit formation programs [reviewed in (Magdalon et al., 2020)].

The complement system has 3 different ways of activation, distinguished by the initiating stimulus: the classical pathway, the lectin pathway and the alternative pathway (Taylor et al., 1998; Sarma and Ward, 2011). In the classical pathway, C1q, as part of the C1 complex (C1qr_2_s_2_), recognizes its target molecule, which can include apoptotic bodies, cellular blebs and antibody-antigen complexes. Following tagging by C1q, the c1r and c1s serine proteases are activated. Activated C1s cleaves C4 and C2, in turn leading to the formation of the C3 convertase C4b2b which cleaves and activates C3, the central effector for complement clearance through all three pathways (Sarma and Ward, 2011). While the classical pathway of the complement system has been implicated in neurodevelopmental processes, for example by producing “eat me” signals for synaptic pruning (Taylor et al., 1998; Sarma and Ward, 2011) (Figure 2), the roles of the lectin and alternative pathways in brain development are less clearly defined and may not be involved.

C1q expression in the brain is punctate and localized to synapses during development (Stevens et al., 2007). Mice deficient for C1q show a significant impairment in synapse elimination, with a higher number of excitatory synaptic connections observed in C1q KO mice (Stevens et al., 2007; Chu et al., 2010). C1q was recently found to recognize and bind to PS, a component of the cellular membrane normally exposed on apoptotic cells that is also found at synapses, suggesting a synergistic mechanism by which synapses could be targeted for pruning by both the PS and complement pathways (Scott-Hewitt et al., 2020; Andoh et al., 2025).

The roles of C3 in neurodevelopment include NPC proliferation, neuronal migration and synaptic pruning (Magdalon et al., 2020). Genetic KO of C3 leads to an increased pool of NPCs in the subventricular zone in early development (Gorelik et al., 2017a). Pharmacological block and genetic deletion of the C3a receptor (C3aR) resulted in opposing phenotypes in the mouse ventricular zone, with a surprising decrease in proliferation in C3aR KO animals compared to WT mice (Coulthard et al., 2018). Therefore, the role of C3 signaling in neuronal proliferation requires further investigation. Deletion of C3 leads to deficits in neuronal migration in the developing mouse cortex, a phenotype that could be rescued by expression of C3a peptide mimics that bind to the C3a receptor (Gorelik et al., 2017a). The functions of C3 in neurodevelopment also involve the complement protein SERPING1, a C1 inhibitor, since its deletion leads to a decrease in NSC proliferation and impeded neuronal migration (Gorelik et al., 2017b).

Many studies have also noted a function of C3 in synaptic pruning, with its loss leading to aberrant pruning in diverse animal models (Lim and Ruthazer, 2021; Schafer et al., 2012). In the developing Xenopus retinotectal system, overexpression of C3 targeted to the presynaptic membrane of retinal ganglion cells resulted in significantly reduced axon arbor length and axon branch complexity compared to control axons (Lim and Ruthazer, 2021). Genetic KO of C3 has the interesting effect of improving place and reversal learning compared to control mice, while reducing probability of release at glutamatergic synapses in the hippocampus (Perez-Alcazar et al., 2014). Non-canonical roles for C3 and C1q include influencing the release of glutamate from astrocytes and neurons and regulating the function of glutamatergic receptor subtypes, including NMDA receptors, AMPA/kainate receptors, and metabotropic glutamate receptors in the plasma membrane (Pittaluga et al., 2025).

Further support for a role of complement pathway components in neurodevelopment comes from evidence of abnormal expression levels and genetic risk in NDDs and psychiatric disorders (Sierra et al., 2022). For instance, GWAS strongly links schizophrenia risk with the MHC locus, where structural variation and copy number diversity drive altered expression levels of C4 (Sekar et al., 2016). In a mouse KO of C4, the segregation of eye-specific laminae in the dLGN was significantly reduced, consistent with impaired synaptic pruning. Additionally, increased levels of C4a, a C4 protein fragment released upon C4 protein activation, have been observed in plasma from schizophrenia patients (Kalinowski et al., 2021). Moreover, iPSC-derived astrocytes from ASD patients showed decreased C4 mRNA and protein expression compared to healthy controls (Mansur et al., 2021). This aligns with the hypothesis that schizophrenia involves excessive synaptic elimination, whereas many forms of ASD, including Fragile X syndrome, are characterized by impaired or insufficient pruning (Faust et al., 2021).

### “Don't eat me” signals

Developmental refinement of neural circuits by synaptic pruning involves the selective sparing of a population of appropriate contacts while targeting inappropriate synapses for elimination by microglia. In contrast to the cell-autonomous regulation of PS localization by membrane-localized flippase and scramblase activity, the pathways leading to microglial activation and target opsonization via complement signaling take place largely in the extracellular space. Thus, positive and negative cues that can distinguish between potential targets of complement-mediated phagocytosis should be present during development and be subject to activity-dependent regulation by neurons. One such candidate is CD47, which is expressed by developing neurons and which inhibits phagocytosis by binding its receptor on microglia, CD47-Signal Regulatory Protein α (SIRPα) receptor (Lehrman et al., 2018). Mice lacking CD47 exhibit increased engulfment of retinogeniculate axon terminals by microglia, as well as reduced synapse numbers in the dLGN. In the developing Xenopus retinotectal projection, complement signaling has been shown to mediate synaptic pruning and RGC arbor remodeling (Lim and Ruthazer, 2021). Xenopus RGCs express a complement C3-interacting protein called Amphibian Regulator of Complement Activation 3 (aRCA3), a putative homolog of mammalian CD46. Overexpression of aRCA3 in RGCs significantly reduces axonal engulfment by microglia and results in axonal arbors that grow to be larger and more extensively branched than controls.

Another promising complement regulatory protein expressed in neurons is CUB and Sushi Multiple Domains 1 (CSMD1) (Baum et al., 2024). CSMD1 is genetically associated with schizophrenia and human stem cell-derived neurons lacking CSMD1 are subject to increased deposition of complement C3. Synaptosomes derived from CSMD1 KO mice are preferentially phagocytosed by microglia *in vitro* compared to WT synaptosomes. Another sushi domain protein, sushi repeat protein X-linked 2 (SRPX2) is a neuronally expressed and secreted complement inhibitor (Cong et al., 2020). SRPX2 directly binds to C1q and blocks its activity, resulting in reduced synapse engulfment by microglia. SRPX2 KO mice have reduced numbers of synapses in the dLGN and somatosensory cortex, consistent with a role in protecting synapses from elimination.

Complement regulatory factors appear to play a key role in protecting certain synapses from pruning in development. As such, they also hold tremendous potential as therapeutic agents to prevent cellular degeneration and synapse loss in disease. Complement receptor 1-related protein Y (CRRY), a complement C3 inhibitor has been found to preserve visual function when expressed in the eye to limit RGC injury in a model of glaucoma (Bosco et al., 2018) and in experimental autoimmune encephalomyelitis (EAE), a model of multiple sclerosis (Werneburg et al., 2020).

Taken together, these findings indicate that complement proteins function in the proper formation and establishment of neuronal circuits by regulating neuronal precursor cell proliferation, neuronal migration, synaptic pruning and activity during development. A better understanding of the mechanisms by which complement signaling targets specific synapses to direct anatomical pruning and functional refinement of developing circuits is likely to provide valuable insights into the etiology of NDDs.

## Conclusions

The formation, fine-tuning, and function of the central nervous system rely extensively on signaling and direct interactions from the innate immune system. This review has outlined just a fraction of the numerous molecular cues and structural interventions that immune mechanisms exert on the developing brain. Notably, pruning of synapses by microglia during circuit refinement has emerged as a fundamental process for healthy neurodevelopment. Genetic linkage studies further underscore this connection, strongly implicating aberrant immune signaling in a range of NDDs and psychiatric illnesses, including ASD and schizophrenia. Elucidating these precise neuroimmune pathways will be invaluable for uncovering new therapeutic targets and advancing our understanding of nervous system development.

## References

[B1] Abarca-Merlin D. M. Martinez-Duran J. A. Medina-Perez J. D. Rodriguez-Santos G. Alvarez-Arellano L. (2024). From immunity to neurogenesis: toll-like receptors as versatile regulators in the nervous system. Int. J. Mol. Sci. 25:5711. doi: 10.3390/ijms25115711 38891900 PMC11171594

[B2] Abbracchio M. P. Burnstock G. Verkhratsky A. Zimmermann H. (2009). Purinergic signalling in the nervous system: an overview. Trends Neurosci. 32, 19–29. doi: 10.1016/j.tins.2008.10.001 19008000

[B3] Ahmed S. Bowers C. Munoz-Martin J. Jamwal S. Sanganahalli B. G. Chen K. . (2026). The microglial TREM2 receptor programs hippocampal development in a mouse model of childhood deprivation. Brain Behav. Immun. 136:106555. doi: 10.1016/j.bbi.2026.106555 41887542 PMC13049556

[B4] Ahn J. Lee J. Kim S. (2015). Interferon-gamma inhibits the neuronal differentiation of neural progenitor cells by inhibiting the expression of Neurogenin2 via the JAK/STAT1 pathway. Biochem. Biophys. Res. Commun. 466, 52–59. doi: 10.1016/j.bbrc.2015.08.104 26325468

[B5] Al-Ayadhi L. Y. Mostafa G. A. (2012). Elevated serum levels of interleukin-17A in children with autism. J. Neuroinflammation. 9:158. doi: 10.1186/1742-2094-9-158 22748016 PMC3410815

[B6] Allan D. Fairlie-Clarke K. J. Elliott C. Schuh C. Barnett S. C. Lassmann H. . (2016). Role of IL-33 and ST2 signalling pathway in multiple sclerosis: expression by oligodendrocytes and inhibition of myelination in central nervous system. Acta Neuropathol. Commun. 4:75. doi: 10.1186/s40478-016-0344-1 27455844 PMC4960877

[B7] Allen N. J. Eroglu C. (2017). Cell biology of astrocyte-synapse interactions. Neuron. 96, 697–708. doi: 10.1016/j.neuron.2017.09.056 29096081 PMC5687890

[B8] Allen N. J. Lyons D. A. (2018). Glia as architects of central nervous system formation and function. Science. 362, 181–185. doi: 10.1126/science.aat0473 30309945 PMC6292669

[B9] Allswede D. M. Buka S. L. Yolken R. H. Torrey E. F. Cannon T. D. (2016). Elevated maternal cytokine levels at birth and risk for psychosis in adult offspring. Schizophr. Res. 172, 41–45. doi: 10.1016/j.schres.2016.02.022 26897476

[B10] Amani M. Houwing D. J. Homberg J. R. Salari A. A. (2021). Perinatal fluoxetine dose-dependently affects prenatal stress-induced neurobehavioural abnormalities, HPA-axis functioning and underlying brain alterations in rat dams and their offspring. Reprod. Toxicol. 104, 27–43. doi: 10.1016/j.reprotox.2021.06.014 34186199

[B11] Andoh M. Shinoda N. Taira Y. Araki T. Kasahara Y. Takeuchi H. . (2025). Nonapoptotic caspase-3 guides C1q-dependent synaptic phagocytosis by microglia. Nature Commun. 16:1. doi: 10.1038/s41467-025-56342-7 39843445 PMC11754728

[B12] Bachstetter A. D. Morganti J. M. Jernberg J. Schlunk A. Mitchell S. H. Brewster K. W. . (2011). Fractalkine and CX3CR1 regulate hippocampal neurogenesis in adult and aged rats. Neurobiol. Aging. 32, 2030–2044. doi: 10.1016/j.neurobiolaging.2009.11.022 20018408 PMC2889032

[B13] Barak B. Feldman N. Okun E. (2014). Toll-like receptors as developmental tools that regulate neurogenesis during development: an update. Front. Neurosci. 8:272. doi: 10.3389/fnins.2014.00272 25221470 PMC4148028

[B14] Barbosa S. Khalfallah O. Forhan A. Galera C. Heude B. Glaichenhaus N. . (2020). Immune activity at birth and later psychopathology in childhood. Brain Behav. Immun. Health. 8:100141. doi: 10.1016/j.bbih.2020.100141 34589885 PMC8474670

[B15] Barron K. D. (1995). The microglial cell. A historical review. J. Neurol. Sci. 34, 57–68. doi: 10.1016/0022-510X(95)00209-K 8847546

[B16] Baum M. L. Wilton D. K. Fox R. G. Carey A. Hsu Y. H. Hu R. . (2024). CSMD1 regulates brain complement activity and circuit development. Brain Behav. Immun. 119, 317–332. doi: 10.1016/j.bbi.2024.03.041 38552925

[B17] Ben-Hur T. Ben-Menachem O. Furer V. Einstein O. Mizrachi-Kol R. Grigoriadis N. . (2003). Effects of proinflammatory cytokines on the growth, fate, and motility of multipotential neural precursor cells. Mol. Cell. Neurosci. 24, 623–631. doi: 10.1016/S1044-7431(03)00218-5 14664813

[B18] Bergon A. Belzeaux R. Comte M. Pelletier F. Herve M. Gardiner E. J. . (2015). CX3CR1 is dysregulated in blood and brain from schizophrenia patients. Schizophr. Res. 168, 434–443. doi: 10.1016/j.schres.2015.08.010 26285829

[B19] Bernardino L. Agasse F. Silva B. Ferreira R. Grade S. Malva J. O. . (2008). Tumor necrosis factor-alpha modulates survival, proliferation, and neuronal differentiation in neonatal subventricular zone cell cultures. Stem Cells 26, 2361–2371. doi: 10.1634/stemcells.2007-0914 18583543

[B20] Bezzi P. Domercq M. Brambilla L. Galli R. Schols D. De Clercq E. . (2001). CXCR4-activated astrocyte glutamate release via TNFalpha: amplification by microglia triggers neurotoxicity. Nat. Neurosci. 4, 702–710. doi: 10.1038/89490 11426226

[B21] Bindokas V. Jordan J. Lee C. Miller R. (1996). Superoxide production in rat hippocampal neurons: selective imaging with hydroethidine. J. Neurosci. 5:16. doi: 10.1523/JNEUROSCI.16-04-01324.1996 8778284 PMC6578569

[B22] Boato F. Hechler D. Rosenberger K. Ludecke D. Peters E. M. Nitsch R. . (2011). Interleukin-1 beta and neurotrophin-3 synergistically promote neurite growth *in vitro*. J. Neuroinflammation. 8:183. doi: 10.1186/1742-2094-8-183 22200088 PMC3275552

[B23] Bobbo V. C. D. Jara C. P. Mendes N. F. Morari J. Velloso L. A. Araujo E. P. . (2019). Interleukin-6 Expression by Hypothalamic Microglia in Multiple Inflammatory Contexts: a systematic review. Biomed Res. Int. 2019:1365210. doi: 10.1155/2019/1365210 31534953 PMC6724433

[B24] Bosco A. Anderson S. R. Breen K. T. Romero C. O. Steele M. R. Chiodo V. A. . (2018). Complement C3-targeted gene therapy restricts onset and progression of neurodegeneration in chronic mouse glaucoma. Mol. Ther. 26, 2379–2396. doi: 10.1016/j.ymthe.2018.08.017 30217731 PMC6171099

[B25] Bowen K. K. Dempsey R. J. Vemuganti R. (2011). Adult interleukin-6 knockout mice show compromised neurogenesis. Neuroreport 22, 126–130. doi: 10.1097/WNR.0b013e3283430a44 21266900

[B26] Burton M. D. Rytych J. L. Freund G. G. Johnson R. W. (2013). Central inhibition of interleukin-6 trans-signaling during peripheral infection reduced neuroinflammation and sickness in aged mice. Brain Behav. Immun. 30, 66–72. doi: 10.1016/j.bbi.2013.01.002 23354002 PMC3641158

[B27] Butovsky O. Ziv Y. Schwartz A. Landa G. Talpalar A. E. Pluchino S. . (2006). Microglia activated by IL-4 or IFN-gamma differentially induce neurogenesis and oligodendrogenesis from adult stem/progenitor cells. Mol. Cell. Neurosci. 31, 149–160. doi: 10.1016/j.mcn.2005.10.006 16297637

[B28] Cao P. Chen C. Liu A. Shan Q. Zhu X. Jia C. . (2021). Early-life inflammation promotes depressive symptoms in adolescence via microglial engulfment of dendritic spines. Neuron. 109, 2573–2589. doi: 10.1016/j.neuron.2021.06.012 34233151

[B29] Casey S. Carter M. Looney A. M. Livingstone V. Moloney G. O'Keeffe G. W. . (2022). Maternal mid-gestation cytokine dysregulation in mothers of children with autism spectrum disorder. J. Autism Dev. Disord. 52, 3919–3932. doi: 10.1007/s10803-021-05271-7 34505185 PMC9349096

[B30] Chen H. Lin W. Zhang Y. Lin L. Chen J. Zeng Y. . (2016). IL-10 promotes neurite outgrowth and synapse formation in cultured cortical neurons after the oxygen-glucose deprivation via JAK1/STAT3 Pathway. Sci. Rep. 6:30459. doi: 10.1038/srep30459 27456198 PMC4960594

[B31] Chen J. Liu X. Zhong Y. (2020). Interleukin-17A: the key cytokine in neurodegenerative diseases. Front. Aging Neurosci. 12:566922. doi: 10.3389/fnagi.2020.566922 33132897 PMC7550684

[B32] Chen Q. Sun Y. Pan N. Long Y. Cao Y. Luo Y. . (2026). Inflammatory cytokine alterations in autism spectrum disorder and attention-deficit/hyperactivity disorder: a systematic review and network meta-analysis. Eur. Child Adolesc. Psychiatr. 13:6. doi: 10.1007/s00787-026-03068-6 42287409

[B33] Chen S. X. Cherry A. Tari P. K. Podgorski K. Kwong Y. K. Haas K. . (2012). The transcription factor MEF2 directs developmental visually driven functional and structural metaplasticity. Cell 151, 41–55. doi: 10.1016/j.cell.2012.08.028 23021214

[B34] Chen Y. Liu J. Zhang S. Gao M. Wang F. Cai M. . (2026). Advances in the multifunctional roles of CX3CL1 in the central nervous system. Front. Aging Neurosci. 18:1696689. doi: 10.3389/fnagi.2026.1696689 41669249 PMC12883738

[B35] Choi G. B. Yim Y. S. Wong H. Kim S. Kim H. Kim S. V. . (2016). The maternal interleukin-17a pathway in mice promotes autism-like phenotypes in offspring. Science. 351, 933–939. doi: 10.1126/science.aad0314 26822608 PMC4782964

[B36] Chu Y. Jin X. Parada I. Pesic A. Stevens B. Barres B. . (2010). Enhanced synaptic connectivity and epilepsy in C1q knockout mice. Proc. Natl. Acad. Sci. USA 107, 7975–7980. doi: 10.1073/pnas.0913449107 20375278 PMC2867906

[B37] Chung W. S. Allen N. J. Eroglu C. (2015). Astrocytes control synapse formation, function, and elimination. Cold Spring Harb. Perspect. Biol. 7:a020370. doi: 10.1101/cshperspect.a020370 25663667 PMC4527946

[B38] Colonna M. Butovsky O. (2017). Microglia function in the central nervous system during health and neurodegeneration. Annu. Rev. Immunol. 35, 441–468. doi: 10.1146/annurev-immunol-051116-052358 28226226 PMC8167938

[B39] Cong Q. Soteros B. M. Wollet M. Kim J. H. Sia G. M. (2020). The endogenous neuronal complement inhibitor SRPX2 protects against complement-mediated synapse elimination during development. Nat. Neurosci. 23, 1067–1078. doi: 10.1038/s41593-020-0672-0 32661396 PMC7483802

[B40] Connolly M. G. Yost O. L. Potter O. V. Giedraitis M. E. Kohman R. A. (2020). Toll-like receptor 4 differentially regulates adult hippocampal neurogenesis in an age- and sex-dependent manner. Hippocampus. 30, 958–969. doi: 10.1002/hipo.23209 32343455 PMC8162993

[B41] Cornfield L. J. Sills M. A. (1991). High affinity interleukin-6 binding sites in bovine hypothalamus. Eur. J. Pharmacol. 202, 113–115. doi: 10.1016/0014-2999(91)90263-p 1786797

[B42] Corriveau R. A. Huh G. S. Shatz C. J. (1998). Regulation of Class I MHC Gene expression in the developing and mature CNS by neural activity. Neuron. 21, 505–520. doi: 10.1016/S0896-6273(00)80562-0 9768838

[B43] Couch A. C. M. Solomon S. Duarte R. R. R. Marrocu A. Sun Y. Sichlinger L. . (2023). Acute IL-6 exposure triggers canonical IL6Ra signaling in hiPSC microglia, but not neural progenitor cells. Brain Behav. Immun. 110, 43–59. doi: 10.1016/j.bbi.2023.02.007 36781081 PMC10682389

[B44] Coulthard L. G. Hawksworth O. A. Conroy J. Lee J. D. Woodruff T. M. (2018). Complement C3a receptor modulates embryonic neural progenitor cell proliferation and cognitive performance. Mol. Immunol. 101, 176–181. doi: 10.1016/j.molimm.2018.06.271 30449309

[B45] Crain J. M. Nikodemova M. Watters J. J. (2009). Expression of P2 nucleotide receptors varies with age and sex in murine brain microglia. J. Neuroinflammation. 6:24. doi: 10.1186/1742-2094-6-24 19706184 PMC2744668

[B46] Crampton S. J. Collins L. M. Toulouse A. Nolan Y. M. O'Keeffe G. W. (2012). Exposure of foetal neural progenitor cells to IL-1beta impairs their proliferation and alters their differentiation - a role for maternal inflammation? J. Neurochem. 120, 964–973. doi: 10.1111/j.1471-4159.2011.07634.x 22192001

[B47] Cserép C. Pósfai B. Lénárt N. Fekete R. László Z. I. Lele Z. . (2020). Microglia monitor and protect neuronal function through specialized somatic purinergic junctions. Science. 367, 528–537. doi: 10.1126/science.aax6752 31831638

[B48] Cserép C. Schwarcz A. D. Posfai B. Laszlo Z. I. Kellermayer A. Kornyei Z. . (2022). Microglial control of neuronal development via somatic purinergic junctions. Cell Rep. 40:111369. doi: 10.1016/j.celrep.2022.111369 36130488 PMC9513806

[B49] Das Sarma J. Ciric B. Marek R. Sadhukhan S. Caruso M. L. Shafagh J. . (2009). Functional interleukin-17 receptor A is expressed in central nervous system glia and upregulated in experimental autoimmune encephalomyelitis. J. Neuroinflammation. 6:14. doi: 10.1186/1742-2094-6-14 19400960 PMC2689857

[B50] Davalos D. Grutzendler J. Yang G. Kim J. V. Zuo Y. Jung S. . (2005). ATP mediates rapid microglial response to local brain injury *in vivo*. Nat. Neurosci. 8, 752–758. doi: 10.1038/nn1472 15895084

[B51] de Cossio Fernandez L. Lacabanne C. Bordeleau M. Castino G. Kyriakakis P. Tremblay M. E. . (2021). Lipopolysaccharide-induced maternal immune activation modulates microglial CX3CR1 protein expression and morphological phenotype in the hippocampus and dentate gyrus, resulting in cognitive inflexibility during late adolescence. Brain Behav. Immun. 97, 440–454. doi: 10.1016/j.bbi.2021.07.025 34343619

[B52] De Felice E. de Andrade Goncalves E. Golia M. T. Gonzalez Ibanez F. Khakpour M. Di Castro M. A. . (2022). Microglial diversity along the hippocampal longitudinal axis impacts synaptic plasticity in adult male mice under homeostatic conditions. J. Neuroinflammation. 19:292. doi: 10.1186/s12974-022-02655-z 36482444 PMC9730634

[B53] Derecki N. C. Cardani A. N. Yang C. H. Quinnies K. M. Crihfield A. Lynch K. R. . (2010). Regulation of learning and memory by meningeal immunity: a key role for IL-4. J. Exp. Med. 207, 1067–1080. doi: 10.1084/jem.20091419 20439540 PMC2867291

[B54] Dutra M. L. Dias P. Freiberger V. Ventura L. Comim C. M. Martins D. F. . (2023). Maternal immune activation induces autism-like behavior and reduces brain-derived neurotrophic factor levels in the hippocampus and offspring cortex of C57BL/6 mice. Neurosci. Lett. 793:136974. doi: 10.1016/j.neulet.2022.136974 36414133

[B55] Eberhardt N. Bergero G. Mazzocco Mariotta Y. L. Aoki M. P. (2022). Purinergic modulation of the immune response to infections. Purinergic Signal. 18, 93–113. doi: 10.1007/s11302-021-09838-y 34997903 PMC8742569

[B56] Erdogan M. A. Turk M. Doganay G. D. Sever I. H. Ozkul B. Sogut I. . (2023). Prenatal SARS-CoV-2 spike protein exposure induces autism-like neurobehavioral changes in male neonatal rats. J. Neuroimmune Pharmacol. 18, 573–591. doi: 10.1007/s11481-023-10089-4 37889404

[B57] Eyo U. B. Wu L. J. (2019). Microglia: lifelong patrolling immune cells of the brain. Prog. Neurobiol. 179:101614. doi: 10.1016/j.pneurobio.2019.04.003 31075285 PMC6599472

[B58] Farina C. Krumbholz M. Giese T. Hartmann G. Aloisi F. Meinl E. . (2005). Preferential expression and function of Toll-like receptor 3 in human astrocytes. J. Neuroimmunol. 159, 12–19. doi: 10.1016/j.jneuroim.2004.09.009 15652398

[B59] Faust T. E. Gunner G. Schafer D. P. (2021). Mechanisms governing activity-dependent synaptic pruning in the developing mammalian CNS. Nat. Rev. Neurosci. 22, 657–673. doi: 10.1038/s41583-021-00507-y 34545240 PMC8541743

[B60] Fialho E. M. S. Veras E. M. Jesus C. M. Gomes L. N. Khouri R. Sousa P. S. . (2023). Maternal Th17 profile after zika virus infection is involved in congenital zika syndrome development in children. Viruses 15:1320. doi: 10.3390/v15061320 37376620 PMC10301288

[B61] Filiano A. J. Xu Y. Tustison N. J. Marsh R. L. Baker W. Smirnov I. . (2016). Unexpected role of interferon-gamma in regulating neuronal connectivity and social behaviour. Nature 535, 425–429. doi: 10.1038/nature18626 27409813 PMC4961620

[B62] Filipello F. Morini R. Corradini I. Zerbi V. Canzi A. Michalski B. . (2018). The microglial innate immune receptor TREM2 is required for synapse elimination and normal brain connectivity. Immunity. 48, 979–991. doi: 10.1016/j.immuni.2018.04.016 29752066

[B63] Gadani S. P. Walsh J. T. Smirnov I. Zheng J. Kipnis J. (2015). The glia-derived alarmin IL-33 orchestrates the immune response and promotes recovery following CNS injury. Neuron. 85, 703–709. doi: 10.1016/j.neuron.2015.01.013 25661185 PMC12064979

[B64] Gadient R. A. Otten U. (1993). Differential expression of interleukin-6 (IL-6) and interleukin-6 receptor (IL-6R) mRNAs in rat hypothalamus. Neurosci. Lett. 153, 13–16. doi: 10.1016/0304-3940(93)90065-S 8510817

[B65] Gadient R. A. Otten U. (1994a). Expression of interleukin-6 (IL-6) and interleukin-6 receptor (IL-6R) mRNAs in rat brain during postnatal development. Brain Res. 637, 10–14. doi: 10.1016/0006-8993(94)91211-4 8180786

[B66] Gadient R. A. Otten U. (1994b). Identification of interleukin-6 (IL-6)-expressing neurons in the cerebellum and hippocampus of normal adult rats. Neurosci. Lett. 182, 243–246. doi: 10.1016/0304-3940(94)90807-9 7715819

[B67] Gallagher D. Norman A. A. Woodard C. L. Yang G. Gauthier-Fisher A. Fujitani M. . (2013). Transient maternal IL-6 mediates long-lasting changes in neural stem cell pools by deregulating an endogenous self-renewal pathway. Cell Stem Cell 13, 564–576. doi: 10.1016/j.stem.2013.10.002 24209760

[B68] Gao C. Jiang J. Tan Y. Chen S. (2023). Microglia in neurodegenerative diseases: mechanism and potential therapeutic targets. Signal Transduct. Target Ther. 8:359. doi: 10.1038/s41392-023-01588-0 37735487 PMC10514343

[B69] Garay P. A. Hsiao E. Y. Patterson P. H. McAllister A. K. (2013). Maternal immune activation causes age- and region-specific changes in brain cytokines in offspring throughout development. Brain Behav. Immun. 31, 54–68. doi: 10.1016/j.bbi.2012.07.008 22841693 PMC3529133

[B70] Geirsdottir L. David E. Keren-Shaul H. Weiner A. Bohlen S. C. Neuber J. . (2019). Cross-species single-cell analysis reveals divergence of the primate microglia program. Cell 179, 1609–1622. doi: 10.1016/j.cell.2019.11.010 31835035

[B71] Giniatullin A. R. Darios F. Shakirzyanova A. Davletov B. Giniatullin R. (2006). SNAP25 is a pre-synaptic target for the depressant action of reactive oxygen species on transmitter release. J. Neurochem. 1:98. doi: 10.1111/j.1471-4159.2006.03997.x 16945102

[B72] Giulian D. Baker T. J. Shih L. C. Lachman L. B. (1986). Interleukin 1 of the central nervous system is produced by ameboid microglia. J. Exp. Med. 164, 594–604. doi: 10.1084/jem.164.2.594 3487617 PMC2188228

[B73] Giulian D. Young D. G. Woodward J. Brown D. C. Lachman L. B. (1988). Interleukin-1 is an astroglial growth factor in the developing brain. J. Neurosci. 8, 709–714. doi: 10.1523/JNEUROSCI.08-02-00709.1988 3257519 PMC6569312

[B74] Goddard C. A. Butts D. A. Shatz C. J. (2007). Regulation of CNS synapses by neuronal MHC class I. Proc. Natl. Acad. Sci. USA 104, 6828–6833. doi: 10.1073/pnas.0702023104 17420446 PMC1871870

[B75] Gomes A. K. S. Dantas R. M. Yokota B. Y. Silva A. Griesi-Oliveira K. Passos-Bueno M. R. . (2022). Interleukin-17a induces neuronal differentiation of induced-pluripotent stem cell-derived neural progenitors from autistic and control subjects. Front. Neurosci. 16:828646. doi: 10.3389/fnins.2022.828646 35360153 PMC8964130

[B76] Gomez Morillas A. Besson V. C. Lerouet D. (2021). Microglia and Neuroinflammation: what place for P2RY12? Int. J. Mol. Sci. 22:1636. doi: 10.3390/ijms22041636 33561958 PMC7915979

[B77] Gonzalez-Madrid E. Rangel-Ramirez M. A. Opazo M. C. Mendez L. Bohmwald K. Bueno S. M. . (2024). Gestational hypothyroxinemia induces ASD-like phenotypes in behavior, proinflammatory markers, and glutamatergic protein expression in mouse offspring of both sexes. Front. Endocrinol. (Lausanne). 15:1381180. doi: 10.3389/fendo.2024.1381180 38752179 PMC11094302

[B78] Gorelik A. Sapir T. Haffner-Krausz R. Olender T. Woodruff T. M. Reiner O. . (2017a). Developmental activities of the complement pathway in migrating neurons. Nature Commun. 8:15096. doi: 10.1038/ncomms15096 PMC5418580 28462915

[B79] Gorelik A. Sapir T. Woodruff T. M. Reiner O. (2017b). Serping1/C1 inhibitor affects cortical development in a cell autonomous and non-cell autonomous manner. Front. Cellular Neurosci. 11:169. doi: 10.3389/fncel.2017.00169 28670268 PMC5472692

[B80] Goshi N. Morgan R. K. Lein P. J. Seker E. A. (2020). A primary neural cell culture model to study neuron, astrocyte, and microglia interactions in neuroinflammation. J. Neuroinflammation. 17:155. doi: 10.1186/s12974-020-01819-z 32393376 PMC7216677

[B81] Griffin W. S. Mrak R. E. (2002). Interleukin-1 in the genesis and progression of and risk for development of neuronal degeneration in Alzheimer's disease. J. Leukoc. Biol. 72, 233–238. doi: 10.1189/jlb.72.2.233 12149413 PMC3835694

[B82] Guedes J. R. Ferreira P. A. Costa J. Laranjo M. Pinto M. J. Reis T. . (2023). IL-4 shapes microglia-dependent pruning of the cerebellum during postnatal development. Neuron. 111, 3435–3449. doi: 10.1016/j.neuron.2023.09.031 37918358

[B83] Guerreiro R. Hardy J. (2013). TREM2 and neurodegenerative disease. N. Engl. J. Med. 369, 1569–1570. doi: 10.1056/NEJMc1306509 24143816

[B84] Gumusoglu S. B. Hing B. W. Q. Chilukuri A. S. S. Dewitt J. J. Scroggins S. M. Stevens H. E. . (2020). Chronic maternal interleukin-17 and autism-related cortical gene expression, neurobiology, and behavior. Neuropsychopharmacology 45, 1008–1017. doi: 10.1038/s41386-020-0640-0 32074626 PMC7162858

[B85] Gunner G. Cheadle L. Johnson K. M. Ayata P. Badimon A. Mondo E. . (2019). Sensory lesioning induces microglial synapse elimination via ADAM10 and fractalkine signaling. Nat. Neurosci. 22, 1075–1088. doi: 10.1038/s41593-019-0419-y 31209379 PMC6596419

[B86] Hama T. Kushima Y. Miyamoto M. Kubota M. Takei N. Hatanaka H. . (1991). Interleukin-6 improves the survival of mesencephalic catecholaminergic and septal cholinergic neurons from postnatal, two-week-old rats in cultures. Neuroscience 40, 445–452. doi: 10.1016/0306-4522(91)90132-8 2027469

[B87] Hama T. Miyamoto M. Tsukui H. Nishio C. Hatanaka H. (1989). Interleukin-6 as a neurotrophic factor for promoting the survival of cultured basal forebrain cholinergic neurons from postnatal rats. Neurosci. Lett. 104, 340–344. doi: 10.1016/0304-3940(89)90600-9 2812549

[B88] Han C. Z. Li R. Z. Hansen E. Trescott S. Fixsen B. R. Nguyen C. T. . (2023). Human microglia maturation is underpinned by specific gene regulatory networks. Immunity 56, 2152–2171. doi: 10.1016/j.immuni.2023.07.016 37582369 PMC10529991

[B89] Han R. T. Vainchtein I. D. Schlachetzki J. C. M. Cho F. S. Dorman L. C. Ahn E. . (2023). Microglial pattern recognition via IL-33 promotes synaptic refinement in developing corticothalamic circuits in mice. J. Exp. Med. 220:e20220605. doi: 10.1084/jem.20220605 36520518 PMC9757845

[B90] Hanisch U. K. Quirion R. (1995). Interleukin-2 as a neuroregulatory cytokine. Brain Res. Brain Res. Rev. 21, 246–284. doi: 10.1016/0165-0173(95)00015-1 8806016

[B91] Hanke M. L. Kielian T. (2011). Toll-like receptors in health and disease in the brain: mechanisms and therapeutic potential. Clin. Sci. (Lond). 121, 367–387. doi: 10.1042/CS20110164 21745188 PMC4231819

[B92] Harrison J. K. Jiang Y. Chen S. Xia Y. Maciejewski D. McNamara R. K. . (1998). Role for neuronally derived fractalkine in mediating interactions between neurons and CX3CR1-expressing microglia. Proc. Natl. Acad. Sci. USA 95, 10896–10901. doi: 10.1073/pnas.95.18.10896 9724801 PMC27992

[B93] He D. Xu H. Zhang H. Tang R. Lan Y. Xing R. . (2022). Disruption of the IL-33-ST2-AKT signaling axis impairs neurodevelopment by inhibiting microglial metabolic adaptation and phagocytic function. Immunity 55, 159–173. doi: 10.1016/j.immuni.2021.12.001 34982959 PMC9074730

[B94] Heinrich P. C. Behrmann I. Haan S. Hermanns H. M. Muller-Newen G. Schaper F. . (2003). Principles of interleukin (IL)-6-type cytokine signalling and its regulation. Biochem. J. 374, 1–20. doi: 10.1042/bj20030407 12773095 PMC1223585

[B95] Heir R. Abbasi Z. Komal P. Altimimi H. F. Franquin M. Moschou D. . (2024). Astrocytes are the source of TNF mediating homeostatic synaptic plasticity. J. Neurosci. 44:e2278222024. doi: 10.1523/JNEUROSCI.2278-22.2024 38395613 PMC10993029

[B96] Hill R. A. Gibbons A. Suwakulsiri W. Taseska A. Darke H. Malhotra A. . (2025). Investigating the impact of severe maternal SARS-CoV-2 infection on infant DNA methylation and neurodevelopment. Mol. Psychiatry. 30, 1976–1984. doi: 10.1038/s41380-024-02808-x 39478169

[B97] Hirano T. Taga T. Nakano N. Yasukawa K. Kashiwamura S. Shimizu K. . (1985). Purification to homogeneity and characterization of human B-cell differentiation factor (BCDF or BSFp-2). Proc. Natl. Acad. Sci. USA. 82, 5490–5494. doi: 10.1073/pnas.82.16.5490 2410927 PMC391148

[B98] Holtman I. R. Glass C. K. Nott A. (2024). “Interpretation of neurodegenerative GWAS risk alleles in microglia and their interplay with other cell types,” in Microglia Physiology, Pathophysiology and Therapeutic Potential, eds. M.-È. Tremblay, and A. Verkhratsky (Switzerland: Springer), 531–44. doi: 10.1007/978-3-031-55529-9 39207711

[B99] Hoshiko M. Arnoux I. Avignone E. Yamamoto N. Audinat E. (2012). Deficiency of the microglial receptor CX3CR1 impairs postnatal functional development of thalamocortical synapses in the barrel cortex. J. Neurosci. 32, 15106–15111. doi: 10.1523/JNEUROSCI.1167-12.2012 23100431 PMC6704837

[B100] Huh G. S. Boulanger L. M. Du H. Riquelme P. A. Brotz T. M. Shatz C. J. . (2000). Functional requirement for Class I MHC in CNS development and plasticity. Science. 290, 2155–2159. doi: 10.1126/science.290.5499.2155 11118151 PMC2175035

[B101] Hung Y. F. Chen C. Y. Shih Y. C. Liu H. Y. Huang C. M. Hsueh Y. P. . (2018). Endosomal TLR3, TLR7, and TLR8 control neuronal morphology through different transcriptional programs. J. Cell Biol. 217, 2727–2742. doi: 10.1083/jcb.201712113 29777026 PMC6080926

[B102] Hymowitz S. G. Filvaroff E. H. Yin J. P. Lee J. Cai L. Risser P. . (2001). IL-17s adopt a cystine knot fold: structure and activity of a novel cytokine, IL-17F, and implications for receptor binding. EMBO J. 20, 5332–5341. doi: 10.1093/emboj/20.19.5332 11574464 PMC125646

[B103] Illes P. Verkhratsky A. Tang Y. (2021). Surveilling microglia dampens neuronal activity: operation of a purinergically mediated negative feedback mechanism. Signal Transduct. Target Ther. 6:160. doi: 10.1038/s41392-021-00586-4 33866328 PMC8053197

[B104] Ishizuka K. Fujita Y. Kawabata T. Kimura H. Iwayama Y. Inada T. . (2017). Rare genetic variants in CX3CR1 and their contribution to the increased risk of schizophrenia and autism spectrum disorders. Transl. Psychiatry. 7:e1184. doi: 10.1038/tp.2017.173 28763059 PMC5611740

[B105] Johansson S. Price J. Modo M. (2008). Effect of inflammatory cytokines on major histocompatibility complex expression and differentiation of human neural stem/progenitor cells. Stem Cells. 26, 2444–2454. doi: 10.1634/stemcells.2008-0116 18635871

[B106] Jones K. L. Croen L. A. Yoshida C. K. Heuer L. Hansen R. Zerbo O. . (2017). Autism with intellectual disability is associated with increased levels of maternal cytokines and chemokines during gestation. Mol. Psychiatry. 22, 273–279. doi: 10.1038/mp.2016.77 27217154 PMC5122473

[B107] Kalinowski A. Liliental J. Anker L. A. Linkovski O. Culbertson C. Hall J. N. . (2021). Increased activation product of complement 4 protein in plasma of individuals with schizophrenia. Transl. Psychiatr. 11:486. doi: 10.1038/s41398-021-01583-5 34552056 PMC8458380

[B108] Kaneko M. Stellwagen D. Malenka R. C. Stryker M. P. (2008). Tumor necrosis factor-alpha mediates one component of competitive, experience-dependent plasticity in developing visual cortex. Neuron. 58, 673–680. doi: 10.1016/j.neuron.2008.04.023 18549780 PMC2884387

[B109] Kaneko N. Kurata M. Yamamoto T. Morikawa S. Masumoto J. (2019). The role of interleukin-1 in general pathology. Inflamm. Regen. 39:12. doi: 10.1186/s41232-019-0101-5 31182982 PMC6551897

[B110] Kany S. Vollrath J. T. Relja B. (2019). Cytokines in inflammatory disease. Int. J. Mol. Sci. 20:6008. doi: 10.3390/ijms20236008 31795299 PMC6929211

[B111] Kerr D. J. Marsillo A. Guariglia S. R. Budylin T. Sadek R. Menkes S. . (2016). Aberrant hippocampal Atp8a1 levels are associated with altered synaptic strength, electrical activity, and autistic-like behavior. Biochim. Biophys. Acta 1862, 1755–1765. doi: 10.1016/j.bbadis.2016.06.005 27287255

[B112] Kiraly B. Csaszar E. Balazsfi D. de Badts C. H. Sviatko K. Posfai B. . (2026). Microglia modulate information processing in the mouse barrel cortex. J. Neurosci. 46:1. doi: 10.1523/JNEUROSCI.0941-25.2026 41735058 PMC13022531

[B113] Kirikae H. Kimura K. Fu J. Sun Z. Wang H. Shibuya H. . (2026). Maternal granulocyte colony-stimulating factor alters synaptic maturation and social behaviors in offspring. Brain Behav. Immun. 135:106534. doi: 10.1016/j.bbi.2026.106534 41825653

[B114] Kirsan F. Z. Dogan O. Yaylaci M. Oztop D. B. (2025). Fractalkine and neuroinflammation in autism spectrum disorder: a novel perspective. Clin. Psychopharmacol. Neurosci. 23, 467–475. doi: 10.9758/cpn.25.1296 40660692 PMC12264666

[B115] Kishimoto T. Akira S. Narazaki M. Taga T. (1995). Interleukin-6 family of cytokines and gp130. Blood. 86, 1243–1254. doi: 10.1182/blood.V86.4.1243.bloodjournal8641243 7632928

[B116] Klann E. Roberson E. D. Knapp L. T. Sweatt J. D. A. (1998). A role for superoxide in protein kinase C activation and induction of long-term potentiation. J. Biol. Chem. 273, 4516–4522. doi: 10.1074/jbc.273.8.4516 9468506

[B117] Kong X. Gong Z. Zhang L. Sun X. Ou Z. Xu B. . (2019). JAK2/STAT3 signaling mediates IL-6-inhibited neurogenesis of neural stem cells through DNA demethylation/methylation. Brain Behav. Immun. 79, 159–173. doi: 10.1016/j.bbi.2019.01.027 30763768

[B118] Kracht L. Borggrewe M. Eskandar S. Brouwer N. de Chuva Sousa Lopes S. M. Laman J. D. . (2020). Human fetal microglia acquire homeostatic immune-sensing properties early in development. Science. 369, 530–537. doi: 10.1126/science.aba5906 32732419

[B119] Lathia J. D. Okun E. Tang S. C. Griffioen K. Cheng A. Mughal M. R. . (2008). Toll-like receptor 3 is a negative regulator of embryonic neural progenitor cell proliferation. J. Neurosci. 28, 13978–139784. doi: 10.1523/JNEUROSCI.2140-08.2008 19091986 PMC2637819

[B120] Le Belle J. E. Orozco N. M. Paucar A. A. Saxe J. P. Mottahedeh J. Pyle A. D. . (2011). Proliferative neural stem cells have high endogenous ROS levels that regulate self-renewal and neurogenesis in a PI3K/Akt-dependant manner. Cell Stem Cell 8, 59–71. doi: 10.1016/j.stem.2010.11.028 21211782 PMC3018289

[B121] Lee R. H. Mills E. A. Schwartz N. Bell M. R. Deeg K. E. Ruthazer E. S. . (2010). Neurodevelopmental effects of chronic exposure to elevated levels of pro-inflammatory cytokines in a developing visual system. Neural Dev. 5:2. doi: 10.1186/1749-8104-5-2 20067608 PMC2819242

[B122] Lehrman E. K. Wilton D. K. Litvina E. Y. Welsh C. A. Chang S. T. Frouin A. . (2018). CD47 Protects synapses from excess microglia-mediated pruning during development. Neuron. 100, 120–134. doi: 10.1016/j.neuron.2018.09.017 30308165 PMC6314207

[B123] Leipzig N. D. Xu C. Zahir T. Shoichet M. S. (2010). Functional immobilization of interferon-gamma induces neuronal differentiation of neural stem cells. J. Biomed. Mater. Res. A. 93, 625–633. doi: 10.1002/jbm.a.32573 19591237

[B124] Li L. Walker T. L. Zhang Y. Mackay E. W. Bartlett P. F. (2010). Endogenous interferon gamma directly regulates neural precursors in the non-inflammatory brain. J. Neurosci. 30, 9038–9050. doi: 10.1523/JNEUROSCI.5691-09.2010 20610738 PMC6632462

[B125] Li T. Chiou B. Gilman C. K. Luo R. Koshi T. Yu D. . (2020). A splicing isoform of GPR56 mediates microglial synaptic refinement via phosphatidylserine binding. EMBO J. 39:e104136. doi: 10.15252/embj.2019104136 32452062 PMC7429740

[B126] Li T. Yu D. Oak H. C. Zhu B. Wang L. Jiang X. . (2021). Phospholipid-flippase chaperone CDC50A is required for synapse maintenance by regulating phosphatidylserine exposure. EMBO J. 40:e107915. doi: 10.15252/embj.2021107915 34585770 PMC8561630

[B127] Li Y. Xu S. Luo L. Yang J. (2024). Role of enzymes capable of transporting phosphatidylserine in brain development and brain diseases. ACS Omega 9, 34243–34249. doi: 10.1021/acsomega.4c05036 39157110 PMC11325426

[B128] Li Z. Jo J. Jia J. M. Lo S. C. Whitcomb D. J. Jiao S. . (2010). Caspase-3 activation via mitochondria is required for long-term depression and AMPA receptor internalization. Cell 141, 859–871. doi: 10.1016/j.cell.2010.03.053 20510932 PMC2909748

[B129] Lim S. H. Park E. You B. Jung Y. Park A. R. Park S. G. . (2013). Neuronal synapse formation induced by microglia and interleukin 10. PLoS ONE. 8:e81218. doi: 10.1371/journal.pone.0081218 24278397 PMC3838367

[B130] Lim T. K. Ruthazer E. S. (2021). Microglial trogocytosis and the complement system regulate axonal pruning *in vivo*. Elife 10:e62167. doi: 10.7554/eLife.62167 33724186 PMC7963485

[B131] Lin S.-S. Tang Y. Illes P. Verkhratsky A. (2021). The Safeguarding microglia: central role for P2Y12 receptors. Front. Pharmacol. 14:11. doi: 10.3389/fphar.2020.627760 33519493 PMC7840491

[B132] Liu H. Y. Hong Y. F. Huang C. M. Chen C. Y. Huang T. N. Hsueh Y. P. . (2013). TLR7 negatively regulates dendrite outgrowth through the Myd88-c-Fos-IL-6 pathway. J. Neurosci. 33, 11479–11493. doi: 10.1523/JNEUROSCI.5566-12.2013 23843519 PMC6618696

[B133] Liu S. Wei S. Sun Y. Xu G. Zhang S. Li J. . (2023). Molecular characteristics functional definitions, and regulatory mechanisms for cross-presentation mediated by the major histocompatibility complex: a comprehensive review. Int. J. Mol. Sci. 25:196. doi: 10.3390/ijms25010196 38203367 PMC10778590

[B134] Liu Z. Wang L. Yu L. Zhao Y. Zhu M. Wang Y. . (2025). Identification of immune cells and circulating inflammatory factors associated with neurodevelopmental disorders by bidirectional Mendelian randomization and mediation analysis. Sci. Rep. 15:12840. doi: 10.1038/s41598-025-98020-0 40229469 PMC11997206

[B135] Lobo-Silva D. Carriche G. M. Castro A. G. Roque S. Saraiva M. (2016). Balancing the immune response in the brain: IL-10 and its regulation. J. Neuroinflammation. 13:297. doi: 10.1186/s12974-016-0763-8 27881137 PMC5121946

[B136] Luo Z. Yang J. (2026). The Role of the IL-33/ST2 axis in nervous system development and diseases. Immunol Cell Biol. 106, 671–681. doi: 10.1111/imcb.70138 42281255 PMC13436154

[B137] Ma L. Li X.-W Zhang S.-J Yang F. Zhu G.-M. Yuan X.-B . (2014). Interleukin-1 beta guides the migration of cortical neurons. J. Neuroinflammation. 11:114. doi: 10.1186/1742-2094-11-114 24950657 PMC4084576

[B138] Ma X. Li X. Wang W. Zhang M. Yang B. Miao Z. . (2022). Phosphatidylserine, inflammation, and central nervous system diseases. Front. Aging Neurosci. 3:14. doi: 10.3389/fnagi.2022.975176 35992593 PMC9382310

[B139] Maciejewski-Lenoir D. Chen S. Feng L. Maki R. Bacon K. B. (1999). Characterization of fractalkine in rat brain cells: migratory and activation signals for CX3CR-1-expressing microglia. J. Immunol. 163, 1628–1635. doi: 10.4049/jimmunol.163.3.1628 10415068

[B140] Magdalon J. Mansur F. Teles E. S. A. L. de Goes V. A. Reiner O. Sertie A. L. . (2020). Complement system in brain architecture and neurodevelopmental disorders. Front. Neurosci. 14:23. doi: 10.3389/fnins.2020.00023 32116493 PMC7015047

[B141] Mandal D. Moitra P. K. Saha S. Basu J. (2002). Caspase 3 regulates phosphatidylserine externalization and phagocytosis of oxidatively stressed erythrocytes. FEBS Lett. 513, 184–188. doi: 10.1016/S0014-5793(02)02294-9 11904147

[B142] Mansur F. Teles E. Silva A. Gomes A. Magdalon J. de Souza J. . (2021). Complement C4 is reduced in iPSC-derived astrocytes of autism spectrum disorder subjects - PubMed. Int. J. Mol. Sci. 22:7579. doi: 10.3390/ijms22147579 34299197 PMC8305914

[B143] Marz P. Herget T. Lang E. Otten U. Rose-John S. (1997). Activation of gp130 by IL-6/soluble IL-6 receptor induces neuronal differentiation. Eur. J. Neurosci. 9, 2765–2773. doi: 10.1111/j.1460-9568.1997.tb01705.x 9517481

[B144] Masi A. Quintana D. S. Glozier N. Lloyd A. R. Hickie I. B. Guastella A. J. . (2015). Cytokine aberrations in autism spectrum disorder: a systematic review and meta-analysis. Mol. Psychiatr. 20, 440–446. doi: 10.1038/mp.2014.59 24934179

[B145] Mastenbroek L. J. M. Kooistra S. M. Eggen B. J. L. Prins J. R. (2024). The role of microglia in early neurodevelopment and the effects of maternal immune activation. Semin. Immunopathol. 46:1. doi: 10.1007/s00281-024-01017-6 38990389 PMC11239780

[B146] Mattei D. Ivanov A. Ferrai C. Jordan P. Guneykaya D. Buonfiglioli A. . (2017). Maternal immune activation results in complex microglial transcriptome signature in the adult offspring that is reversed by minocycline treatment. Transl. Psychiatry. 7:e1120. doi: 10.1038/tp.2017.80 28485733 PMC5534948

[B147] Mazaheri F. Breus O. Durdu S. Haas P. Wittbrodt J. Gilmour D. . (2014). Distinct roles for BAI1 and TIM-4 in the engulfment of dying neurons by microglia. Nat. Commun. 5:4046. doi: 10.1038/ncomms5046 24898390

[B148] Mendez-Salcido F. A. Torres-Flores M. I. Ordaz B. Pena-Ortega F. (2022). Abnormal innate and learned behavior induced by neuron-microglia miscommunication is related to CA3 reconfiguration. Glia 70, 1630–1651. doi: 10.1002/glia.24185 35535571

[B149] Mildner A. Huang H. Radke J. Stenzel W. Priller J. (2017). P2Y_12_ receptor is expressed on human microglia under physiological conditions throughout development and is sensitive to neuroinflammatory diseases. Glia 65, 375–387. doi: 10.1002/glia.23097 27862351

[B150] Mo M. Eyo U. B. Xie M. Peng J. Bosco D. B. Umpierre A. D. . (2019). Microglial P2Y12 receptor regulates seizure-induced neurogenesis and immature neuronal projections. J. Neurosci. 39, 9453–9464. doi: 10.1523/JNEUROSCI.0487-19.2019 31597724 PMC6867812

[B151] Mordelt A. Schuurmans I. M. E. Scheefhals N. Hommersom M. P. Slottje K. Mast K. . (2026). Development and validation of a long-term co-maturation protocol for human stem cell-derived microglia and neuronal networks. Stem Cell Rep. 21:103053. doi: 10.1016/j.stemcr.2026.103053 42624101 PMC13555563

[B152] Mousa A. Seiger A. Kjaeldgaard A. Bakhiet M. (1999). Human first trimester forebrain cells express genes for inflammatory and anti-inflammatory cytokines. Cytokine 11, 55–60. doi: 10.1006/cyto.1998.0381 10080879

[B153] Murakami Y. Imamura Y. Kasahara Y. Yoshida C. Momono Y. Fang K. . (2021). The effects of maternal interleukin-17A on social behavior, cognitive function, and depression-like behavior in mice with altered kynurenine metabolites. Int. J. Tryptophan Res. 14:11786469211026639. doi: 10.1177/11786469211026639 34262289 PMC8243115

[B154] Nakaso K. (2024). Roles of microglia in neurodegenerative diseases. Yonago Acta. Med. 67, 1–8. doi: 10.33160/yam.2024.02.001 38380436 PMC10867232

[B155] Nelson P. A. Sage J. R. Wood S. C. Davenport C. M. Anagnostaras S. G. Boulanger L. M. M. H. C. . (2013). Class I immune proteins are critical for hippocampus-dependent memory and gate NMDAR-dependent hippocampal long-term depression. Learning and Memory. 1:20. doi: 10.1101/lm.031351.113 23959708 PMC3744042

[B156] Neniskyte U. Kuliesiute U. Vadisiute A. Jevdokimenko K. Coletta L. Deivasigamani S. . (2023). Phospholipid scramblase Xkr8 is required for developmental axon pruning via phosphatidylserine exposure. EMBO J. 42:e111790. doi: 10.15252/embj.2022111790 37211968 PMC10350823

[B157] Network Pathway Analysis Subgroup of Psychiatric Genomics C. (2015). Psychiatric genome-wide association study analyses implicate neuronal, immune and histone pathways. Nat. Neurosci. 18, 199–209. doi: 10.1038/nn.3922 25599223 PMC4378867

[B158] Neumann H. Schmidt H. Cavalie A. Jenne D. Wekerle H. (1997). Major histocompatibility complex (MHC) class I gene expression in single neurons of the central nervous system: differential regulation by interferon (IFN)-gamma and tumor necrosis factor (TNF)-alpha. J. Exp. Med. 185, 305–316. doi: 10.1084/jem.185.2.305 9016879 PMC2196130

[B159] Nguyen P. T. Dorman L. C. Pan S. Vainchtein I. D. Han R. T. Nakao-Inoue H. . (2020). Microglial remodeling of the extracellular matrix promotes synapse plasticity. Cell 182, 388–403. doi: 10.1016/j.cell.2020.05.050 32615087 PMC7497728

[B160] Nimmerjahn A. Kirchhoff F. Helmchen F. (2005). Resting microglial cells are highly dynamic surveillants of brain parenchyma *in vivo*. Science. 308, 1314–1318. doi: 10.1126/science.1110647 15831717

[B161] Niwa H. Burdon T. Chambers I. Smith A. (1998). Self-renewal of pluripotent embryonic stem cells is mediated via activation of STAT3. Genes Dev. 12, 2048–2060. doi: 10.1101/gad.12.13.2048 9649508 PMC316954

[B162] O'Donnell L. A. Henkins K. M. Kulkarni A. Matullo C. M. Balachandran S. Pattisapu A. K. . (2015). Interferon gamma induces protective non-canonical signaling pathways in primary neurons. J. Neurochem. 135, 309–322. doi: 10.1111/jnc.13250 26190522 PMC4809142

[B163] Ohsawa K. Irino Y. Sanagi T. Nakamura Y. Suzuki E. Inoue K. . (2010). P2Y1_2_ receptor-mediated integrin-beta1 activation regulates microglial process extension induced by ATP. Glia 58, 790–801. doi: 10.1002/glia.20963 20091784

[B164] Okun E. Griffioen K. Barak B. Roberts N. J. Castro K. Pita M. A. . (2010). Toll-like receptor 3 inhibits memory retention and constrains adult hippocampal neurogenesis. Proc. Natl. Acad. Sci. USA 107, 15625–15630. doi: 10.1073/pnas.1005807107 20713712 PMC2932590

[B165] O'Shea J. J. Murray P. J. (2008). Cytokine signaling modules in inflammatory responses. Immunity 28, 477–487. doi: 10.1016/j.immuni.2008.03.002 18400190 PMC2782488

[B166] Oswald M. C. W. Brooks P. S. Zwart M. F. Mukherjee A. West R. J. H. Giachello C. N. G. . (2018b). Reactive oxygen species regulate activity-dependent neuronal plasticity in Drosophila. eLife 7:e39393. doi: 10.7554/eLife.39393 30540251 PMC6307858

[B167] Oswald M. C. W. Garnham N. Sweeney S. T. Landgraf M. (2018a). Regulation of neuronal development and function by ROS. FEBS Lett. 592, 679–691. doi: 10.1002/1873-3468.12972 29323696 PMC5888200

[B168] Paolicelli R. C. Bisht K. Tremblay M. E. (2014). Fractalkine regulation of microglial physiology and consequences on the brain and behavior. Front. Cell. Neurosci. 8:129. doi: 10.3389/fncel.2014.00129 24860431 PMC4026677

[B169] Paolicelli R. C. Bolasco G. Pagani F. Maggi L. Scianni M. Panzanelli P. . (2011). Synaptic pruning by microglia is necessary for normal brain development. Science. 333, 1456–1458. doi: 10.1126/science.1202529 21778362

[B170] Paolicelli R. C. Ferretti M. T. (2017). Function and dysfunction of microglia during brain development: consequences for synapses and neural circuits. Front. Synaptic. Neurosci. 9:9. doi: 10.3389/fnsyn.2017.00009 28539882 PMC5423952

[B171] Park J. Choi Y. Jung E. Lee S. H. Sohn J. W. Chung W. S. . (2021). Microglial MERTK eliminates phosphatidylserine-displaying inhibitory post-synapses. EMBO J. 40:e107121. doi: 10.15252/embj.2020107121 34013588 PMC8327958

[B172] Park S. Y. Kang M. J. Han J. S. (2018). Interleukin-1 beta promotes neuronal differentiation through the Wnt5a/RhoA/JNK pathway in cortical neural precursor cells. Mol. Brain. 11:39. doi: 10.1186/s13041-018-0383-6 29973222 PMC6033214

[B173] Pasciuto E. Burton O. T. Roca C. P. Lagou V. Rajan W. D. Theys T. . (2020). Microglia require CD4 T cells to complete the fetal-to-adult transition. Cell 182, 625–640. doi: 10.1016/j.cell.2020.06.026 32702313 PMC7427333

[B174] Peet G. Bennett F. C. Bennett M. L. (2020). Please eat (only part) of me: synaptic phosphatidylserine cues microglia to feast: two new studies identify how a common apoptotic cell flag is used to sculpt neural circuits. EMBO J. 39:e105924. doi: 10.15252/embj.2020105924 32705698 PMC7429477

[B175] Pekkoc Uyanik K. C. Kalayci Yigin A. Dogangun B. Seven M. (2022). Evaluation of IL1B rs1143634 and IL6 rs1800796 polymorphisms with autism spectrum disorder in the turkish children. Immunol. Invest. 51, 766–777. doi: 10.1080/08820139.2020.1870489 33432850

[B176] Peng J. Liu Y. Umpierre A. D. Xie M. Tian D. S. Richardson J. R. . (2019). Microglial P2Y12 receptor regulates ventral hippocampal CA1 neuronal excitability and innate fear in mice. Mol. Brain. 12:71. doi: 10.1186/s13041-019-0492-x 31426845 PMC6700820

[B177] Peng J. J. Lin S. H. Liu Y. T. Lin H. C. Li T. N. Yao C. K. A. . (2019). Circuit-dependent ROS feedback loop mediates glutamate excitotoxicity to sculpt the Drosophila motor system. Elife 8:e47372. doi: 10.7554/eLife.47372 PMC6682402 31318331

[B178] Perez-Alcazar M. Daborg J. Stokowska A. Wasling P. Bjorefeldt A. Kalm M. . (2014). Altered cognitive performance and synaptic function in the hippocampus of mice lacking C3. Exp. Neurol. 253, 154–164. doi: 10.1016/j.expneurol.2013.12.013 24378428

[B179] Perez-Asensio F. J. Perpina U. Planas A. M. Pozas E. (2013). Interleukin-10 regulates progenitor differentiation and modulates neurogenesis in adult brain. J. Cell Sci. 126, 4208–4219. doi: 10.1242/jcs.127803 23843621

[B180] Petitto J. M. Meola D. Huang Z. (2012). Interleukin-2 and the brain: dissecting central versus peripheral contributions using unique mouse models. Methods Mol. Biol. 934, 301–311. doi: 10.1007/978-1-62703-071-7_15 22933152

[B181] Pittaluga A. Torre V. Olivero G. Rosenwasser N. Taddeucci A. (2025). Non-canonical roles of complement in the CNS: from synaptic organizer to presynaptic modulator of glutamate transmission. Curr. Neuropharmacol. 14:23. doi: 10.2174/011570159X327960240823065729 39817397 PMC12163473

[B182] Polan M. B. Pastore M. T. Steingass K. Hashimoto S. Thrush D. L. Pyatt R. . (2014). Neurodevelopmental disorders among individuals with duplication of 4p13 to 4p12 containing a GABAA receptor subunit gene cluster. Eur. J. Hum. Genet. 22, 105–109. doi: 10.1038/ejhg.2013.99 23695283 PMC3865421

[B183] Popova G. Soliman S. S. Kim C. N. Keefe M. G. Hennick K. M. Jain S. . (2021). Human microglia states are conserved across experimental models and regulate neural stem cell responses in chimeric organoids. Cell Stem Cell 28, 2153–2166. doi: 10.1016/j.stem.2021.08.015 34536354 PMC8642295

[B184] Porter A. G. Jänicke R. U. Porter A. G. Jänicke R. U. (1999). Emerging roles of caspase-3 in apoptosis. Cell Death Different. 6:2. doi: 10.1038/sj.cdd.4400476 10200555

[B185] Posfai B. Szabadits E. Cserep C. Vida S Schwarcz A. D. Fekete R. . (2026). P2Y12 Receptor function governs microglial surveillance and cell-cell interactions in the cerebral cortex. Glia 74:e70109. doi: 10.1002/glia.70109 41331281

[B186] Pratt L. Ni L. Ponzio N. M. Jonakait G. M. (2013). Maternal inflammation promotes fetal microglial activation and increased cholinergic expression in the fetal basal forebrain: role of interleukin-6. Pediatr. Res. 74, 393–401. doi: 10.1038/pr.2013.126 23877071

[B187] Qian C. Cao X. Hoffmann J. A. (2026). Toll-like receptors in innate immunity and inflammation: from fundamental biology to clinic insights. Immun. Inflamm. 2:22. doi: 10.1007/s44466-026-00040-6 42052154 PMC13111532

[B188] Raiders S. Black E. C. Bae A. MacFarlane S. Klein M. Shaham S. . (2021). Glia actively sculpt sensory neurons by controlled phagocytosis to tune animal behavior. Elife 10:e63532. doi: 10.7554/eLife.63532 33759761 PMC8079151

[B189] Ramirez J. J. Hardin E. J. Sakers K. Kim J. Colon Ortiz C. Savage J. T. . (2026). Astrocyte-microglia crosstalk through Hevin and Toll-like receptor signaling controls developmental thalamocortical synapse refinement. Neuron. 114, 1433–1453. doi: 10.1016/j.neuron.2025.12.028 41720089 PMC13242099

[B190] Rio-Hortega P. (1919a). El “Tercer Elemento” de los centros nerviosos. I. La microglia en estado normal. Boletin de la Sociedad Espanol de Biologia VIII, 67–82.

[B191] Rio-Hortega P. (1919b). El “Tercer Elemento de los Centros Nerviosos”. II. Intervencion de la microglia en los procesos patologicos (Celulas en bastoncito y cuerpos granuloadiposos). Boletin de la Sociedad Espanol de Biologia VIII, 91–103.

[B192] Rio-Hortega P. (1919c). El “Tercer Elemento” de los centros nerviosos. IIIl. Naturaleza probable de la microglia. Boletin de la Sociedad Espanol de Biologia VIII, 108–121.

[B193] Rio-Hortega P. (1919d). El “Tercer Elemento de los centros nerviosos”. IV. Poder fagocitario y movilidad de la microglia. Boletin de la Sociedad Espanol de Biologia VIII, 154–171.

[B194] Rodrigues R. J. Marques J. M. Cunha R. A. (2019). Purinergic signalling and brain development. Semin. Cell Dev. Biol. 95, 34–41. doi: 10.1016/j.semcdb.2018.12.001 30529149

[B195] Rogers J. T. Morganti J. M. Bachstetter A. D. Hudson C. E. Peters M. M. Grimmig B. A. . (2011). CX3CR1 deficiency leads to impairment of hippocampal cognitive function and synaptic plasticity. J. Neurosci. 31, 16241–16250. doi: 10.1523/JNEUROSCI.3667-11.2011 22072675 PMC3236509

[B196] Rolls A. Shechter R. London A. Ziv Y. Ronen A. Levy R. . (2007). Toll-like receptors modulate adult hippocampal neurogenesis. Nat. Cell Biol. 9, 1081–1088. doi: 10.1038/ncb1629 17704767

[B197] Sallam D. E. Shaker Y. S. Mostafa G. A. El-Hossiny R. M. Taha S. I. Ahamed M. . (2024). Evaluation of serum interleukin-17 A and interleukin-22 levels in pediatric patients with autism spectrum disorder: a pilot study. BMC Pediatr. 24:18. doi: 10.1186/s12887-023-04484-2 38183030 PMC10768424

[B198] Salvador Y. M. U. (2022). “Purinergic system in immune response,” in Purinergic System, eds. M. D. Bagatini, and A. M. Carnoma-Ribiero (Brazil: IntechOpen).

[B199] Santello M. Bezzi P. Volterra A. (2011). TNFα controls glutamatergic gliotransmission in the hippocampal dentate gyrus. Neuron. 69, 988–1001. doi: 10.1016/j.neuron.2011.02.003 21382557

[B200] Sarma J. V. Ward P. A. (2011). The complement system. Cell Tissue Res. 343, 227–235. doi: 10.1007/s00441-010-1034-0 20838815 PMC3097465

[B201] Sasaki T. Tome S. Takei Y. (2020). Intraventricular IL-17A administration activates microglia and alters their localization in the mouse embryo cerebral cortex. Mol. Brain. 13:93. doi: 10.1186/s13041-020-00635-z 32546246 PMC7298827

[B202] Schafer D. P. Lehrman E. K. Kautzman A. G. Koyama R. Mardinly A. R. Yamasaki R. . (2012). Microglia sculpt postnatal neural circuits in an activity and complement-dependent manner. Neuron. 74, 691–705. doi: 10.1016/j.neuron.2012.03.026 22632727 PMC3528177

[B203] Schepanski S. Ngoumou G. B. Buss C. Seifert G. (2025). Assessing *in-vitro* models for microglial development and fetal programming: a critical review. Front. Immunol. 16:1538920. doi: 10.3389/fimmu.2025.1538920 39944696 PMC11814449

[B204] Schobitz B. de Kloet E. R. Sutanto W. Holsboer F. (1993). Cellular localization of interleukin 6 mRNA and interleukin 6 receptor mRNA in rat brain. Eur. J. Neurosci. 5, 1426–1435. doi: 10.1111/j.1460-9568.1993.tb00210.x 8287192

[B205] Scianni M. Antonilli L. Chece G. Cristalli G. Di Castro M. A. Limatola C. . (2013). Fractalkine (CX3CL1) enhances hippocampal N-methyl-D-aspartate receptor (NMDAR) function via D-serine and adenosine receptor type A2 (A2AR) activity. J. Neuroinflammation. 10:108. doi: 10.1186/1742-2094-10-108 23981568 PMC3765929

[B206] Scott-Hewitt N. Perrucci F. Morini R. Erreni M. Mahoney M. Witkowska A. . (2020). Local externalization of phosphatidylserine mediates developmental synaptic pruning by microglia. EMBO J. 39:e105380. doi: 10.15252/embj.2020105380 32657463 PMC7429741

[B207] Sekar A. Bialas A. R. Rivera d. e. Davis H. Hammond A. Kamitaki T. R. N. . (2016). Schizophrenia risk from complex variation of complement component 4. Nature 530, 177–183. doi: 10.1038/nature16549 26814963 PMC4752392

[B208] Seong K. J. Choi S. Lee H. G. Rhee J. H. Lee J. H. Koh J. T. . (2022). Toll-Like Receptor 5 promotes the neurogenesis from embryonic stem cells and adult hippocampal neural stem cells in Mice. Stem Cells. 40, 303–317. doi: 10.1093/stmcls/sxab025 35304896

[B209] Shacham-Silverberg V. Sar Shalom H. Goldner R. Golan-Vaishenker Y. Gurwicz N. Gokhman I. . (2018). Phosphatidylserine is a marker for axonal debris engulfment but its exposure can be decoupled from degeneration. Cell Death and Disease 9:11. doi: 10.1038/s41419-018-1155-z 30389906 PMC6214901

[B210] Shechter R. Ronen A. Rolls A. London A. Bakalash S. Young M. J. . (2008). Toll-like receptor 4 restricts retinal progenitor cell proliferation. J. Cell Biol. 183, 393–400. doi: 10.1083/jcb.200804010 18981228 PMC2575781

[B211] Sierra A. de Castro F. Del Rio-Hortega J. Rafael Iglesias-Rozas J. Garrosa M. Kettenmann H. . (2016). The “Big-Bang” for modern glial biology: translation and comments on Pio del Rio-Hortega 1919 series of papers on microglia. Glia 64, 1801–1840. doi: 10.1002/glia.23046 27634048

[B212] Sierra D. P. Tripathi A. Pillai A. (2022). Dysregulation of complement system in neuropsychiatric disorders: a mini review. Biomark Neuropsychiatr. 7:100056. doi: 10.1016/j.bionps.2022.100056 37123465 PMC10136364

[B213] Sipe G. O. Lowery R. L. Tremblay M.-È. Kelly E. A. Lamantia C. E. Majewska A. K. . (2016). Microglial P2Y12 is necessary for synaptic plasticity in mouse visual cortex. Nat. Commun. 7:1. doi: 10.1038/ncomms10905 26948129 PMC4786684

[B214] Smail M. A. Lenz K. M. (2024). Developmental functions of microglia: impact of psychosocial and physiological early life stress. Neuropharmacology. 258:110084. doi: 10.1016/j.neuropharm.2024.110084 39025401 PMC12051134

[B215] Solek C. M. Farooqi N. Brake A. I. Kesner P. Schohl A. Antel J. P. . (2021). Early inflammation dysregulates neuronal circuit formation *in vivo* via upregulation of IL-1beta. J. Neurosci. 41, 6353–6366. doi: 10.1523/JNEUROSCI.2159-20.2021 34103360 PMC8287996

[B216] Solek C. M. Farooqi N. Verly M. Lim T. K. Ruthazer E. S. (2018). Maternal immune activation in neurodevelopmental disorders. Dev. Dyn. 247, 588–619. doi: 10.1002/dvdy.24612 29226543

[B217] Song J. H. Wang C. X. Song D. K. Wang P. Shuaib A. Hao C. . (2005). Interferon γ induces neurite outgrowth by up-regulation of p35 neuron-specific cyclin-dependent kinase 5 activator via activation of ERK1/2 pathway. J. Biol. Chem. 280, 12896–12901. doi: 10.1074/jbc.M412139200 15695523

[B218] Sterneck E. Kaplan D. R. Johnson P. F. (1996). Interleukin-6 induces expression of peripherin and cooperates with Trk receptor signaling to promote neuronal differentiation in PC12 cells. J. Neurochem. 67, 1365–1374. doi: 10.1046/j.1471-4159.1996.67041365.x 8858917

[B219] Stevens B. Allen N. J. Vazquez L. E. Howell G. R. Christopherson K. S. Nouri N. . (2007). The classical complement cascade mediates CNS synapse elimination. Cell. 131, 1164–1178. doi: 10.1016/j.cell.2007.10.036 18083105

[B220] Striedinger K. Scemes E. (2008). Interleukin-1beta affects calcium signaling and *in vitro* cell migration of astrocyte progenitors. J. Neuroimmunol. 196, 116–123. doi: 10.1016/j.jneuroim.2008.03.014 18462808 PMC2453308

[B221] Sulistio Y. A. Lee H. K. Jung S. J. Heese K. (2018). Interleukin-6-mediated induced pluripotent stem cell (iPSC)-derived neural differentiation. Mol. Neurobiol. 55, 3513–3522. doi: 10.1007/s12035-017-0594-3 28509081

[B222] Sung H. Y. Chen W. Y. Huang H. T. Wang C. Y. Chang S. B. Tzeng S. F. . (2019). Down-regulation of interleukin-33 expression in oligodendrocyte precursor cells impairs oligodendrocyte lineage progression. J. Neurochem. 150, 691–708. doi: 10.1111/jnc.14788 31165473

[B223] Suyama S. Sunabori T. Kanki H. Sawamoto K. Gachet C. Koizumi S. . (2012). Purinergic signaling promotes proliferation of adult mouse subventricular zone cells. J. Neurosci. 32, 9238–9247. doi: 10.1523/JNEUROSCI.4001-11.2012 22764232 PMC6622243

[B224] Suzuki K. Matsuzaki H. Iwata K. Kameno Y. Shimmura C. Kawai S. . (2011). Plasma cytokine profiles in subjects with high-functioning autism spectrum disorders. PLoS ONE 6:e20470. doi: 10.1371/journal.pone.0020470 21647375 PMC3103577

[B225] Szabo D. Otrokocsi L. Sperlagh B. (2025). The role of maternal infections in neurodevelopmental psychiatric disorders: focus on the P2X7/NLRP3/IL-1beta signalling pathway. J. Neuroinflammation. 22:192. doi: 10.1186/s12974-025-03509-0 40713568 PMC12298051

[B226] Tanabe S. Yamashita T. (2018). B-1a lymphocytes promote oligodendrogenesis during brain development. Nat. Neurosci. 21, 506–516. doi: 10.1038/s41593-018-0106-4 29507409

[B227] Tarozzo G. Bortolazzi S. Crochemore C. Chen S. C. Lira A. S. Abrams J. S. . (2003). Fractalkine protein localization and gene expression in mouse brain. J. Neurosci. Res. 73, 81–88. doi: 10.1002/jnr.10645 12815711

[B228] Tarozzo G. Campanella M. Ghiani M. Bulfone A. Beltramo M. (2002). Expression of fractalkine and its receptor, CX3CR1, in response to ischaemia-reperfusion brain injury in the rat. Eur. J. Neurosci. 15, 1663–1668. doi: 10.1046/j.1460-9568.2002.02007.x 12059974

[B229] Taylor P. Botto M. Walport M. (1998). The complement system. Curr. Biol. 8, R259–R261. doi: 10.1016/S0960-9822(98)70167-8 9550710

[B230] Ueno M. Fujita Y. Tanaka T. Nakamura Y. Kikuta J. Ishii M. . (2013). Layer V cortical neurons require microglial support for survival during postnatal development. Nat. Neurosci. 16, 543–551. doi: 10.1038/nn.3358 23525041

[B231] Utz S. G. See P. Mildenberger W. Thion M. S. Silvin A. Lutz M. . (2020). Early fate defines microglia and non-parenchymal brain macrophage development. Cell 181, 557–573. doi: 10.1016/j.cell.2020.03.021 32259484

[B232] Vainchtein I. D. Chin G. Cho F. S. Kelley K. W. Miller J. G. Chien E. C. . (2018). Astrocyte-derived interleukin-33 promotes microglial synapse engulfment and neural circuit development. Science. 359, 1269–1273. doi: 10.1126/science.aal3589 29420261 PMC6070131

[B233] Valanne S. Vesala L. Maasdorp M. K. Salminen T. S. Ramet M. (2022). The Drosophila toll pathway in innate immunity: from the core pathway toward effector functions. J. Immunol. 209, 1817–1825. doi: 10.4049/jimmunol.2200476 36426939

[B234] Valanne S. Wang J. H. Ramet M. (2011). The *Drosophila* Toll signaling pathway. J. Immunol. 186, 649–656. doi: 10.4049/jimmunol.1002302 21209287

[B235] Vallieres L. Rivest S. (1997). Regulation of the genes encoding interleukin-6, its receptor, and gp130 in the rat brain in response to the immune activator lipopolysaccharide and the proinflammatory cytokine interleukin-1β. J. Neurochem. 69, 1668–1683. doi: 10.1046/j.1471-4159.1997.69041668.x 9326296

[B236] Verma D. Dhar S. Malemnganba T. Sharma S. Kar S. Prajapati V. K. T. L. R. . (2026). Ligands as immunomodulators: mechanistic insights and therapeutic potential in infectious and inflammatory diseases. Mol. Biol. Rep. 53:856. doi: 10.1007/s11033-026-12053-w 42213280

[B237] Verma R. Kim J. Y. (2016). 1,25-Dihydroxyvitamin D_3_ Facilitates M2 Polarization and Upregulates TLR10 Expression on Human Microglial Cells. *Neuroimmunomodulation*. 23, 75–80. doi: 10.1159/000444300 26999663

[B238] Vezzani A. Viviani B. (2015). Neuromodulatory properties of inflammatory cytokines and their impact on neuronal excitability. Neuropharmacology. 96, 70–82. doi: 10.1016/j.neuropharm.2014.10.027 25445483

[B239] Voineagu I. Eapen V. (2013). Converging pathways in autism spectrum disorders: interplay between synaptic dysfunction and immune responses. Front. Hum. Neurosci. 7:738. doi: 10.3389/fnhum.2013.00738 24223544 PMC3819618

[B240] Wang Q. Shi N.-R. Lv P. Liu J. Zhang J.-Z. Deng B.-L. . (2022). P2Y12 receptor gene polymorphisms are associated with epilepsy. Purinergic Signalling 19, 155–162. doi: 10.1007/s11302-022-09848-4 35175489 PMC9984642

[B241] Watson A. E. S. Goodkey K. Footz T. Voronova A. (2020). Regulation of CNS precursor function by neuronal chemokines. Neurosci. Lett. 715:134533. doi: 10.1016/j.neulet.2019.134533 31629772

[B242] Wei H. Alberts I. Brain Li X. (2013). IL-6 and autism. Neuroscience 252, 320–325. doi: 10.1016/j.neuroscience.2013.08.025 23994594

[B243] Werneburg S. Jung J. Kunjamma R. B. Ha S. K. Luciano N. J. Willis C. M. . (2020). Targeted complement inhibition at synapses prevents microglial synaptic engulfment and synapse loss in demyelinating disease. Immunity 52, 167–182. doi: 10.1016/j.immuni.2019.12.004 31883839 PMC6996144

[B244] Whitelaw B. S. Stoessel M. B. Majewska A. K. (2022). Movers and shakers: microglial dynamics and modulation of neural networks. Glia 71, 1575–1591. doi: 10.1002/glia.24323 36533844 PMC10729610

[B245] Wicher G. Husic E. Nilsson G. Forsberg-Nilsson K. (2013). Developmental expression of IL-33 in the mouse brain. Neurosci. Lett. 555, 171–176. doi: 10.1016/j.neulet.2013.09.046 24076135

[B246] Wilson C. Núñez M. T. González-Billault C. (2015). Contribution of NADPH oxidase to the establishment of hippocampal neuronal polarity in culture. J. Cell Sci. 15:128. doi: 10.1242/jcs.168567 26101350

[B247] Winter A. N. Subbarayan M. S. Grimmig B. Weesner J. A. Moss L. Peters M. . (2020). Two forms of CX3CL1 display differential activity and rescue cognitive deficits in CX3CL1 knockout mice. J. Neuroinflammation. 17:157. doi: 10.1186/s12974-020-01828-y 32410624 PMC7227354

[B248] Wong G. Goldshmit Y. Turnley A. M. (2004). Interferon-gamma but not TNFα promotes neuronal differentiation and neurite outgrowth of murine adult neural stem cells. Exp. Neurol. 187, 171–177. doi: 10.1016/j.expneurol.2004.01.009 15081598

[B249] Wood L. B. Singer A. C. (2025). Neurons as immunomodulators: from rapid neural activity to prolonged regulation of cytokines and microglia. Annu. Rev. Biomed. Eng. 27, 55–72. doi: 10.1146/annurev-bioeng-110122-120158 39805040 PMC12486157

[B250] Wu Z. P. Washburn L. Bilousova T. V. Boudzinskaia M. Escande-Beillard N. Querubin J. . (2011). Enhanced neuronal expression of major histocompatibility complex class I leads to aberrations in neurodevelopment and neurorepair. J. Neuroimmunol. 232, 8–16. doi: 10.1016/j.jneuroim.2010.09.009 20950866 PMC5776042

[B251] Yan H. Q. Banos M. A. Herregodts P. Hooghe R. Hooghe-Peters E. L. (1992). Expression of interleukin (IL)-1β, IL-6 and their respective receptors in the normal rat brain and after injury. Eur. J. Immunol. 22, 2963–2971. doi: 10.1002/eji.1830221131 1425920

[B252] Yang Y. Bazhin A. V. Werner J. Karakhanova S. (2013). Reactive oxygen species in the immune system. Int. Rev. Immunol. 1:32. doi: 10.3109/08830185.2012.755176 23617726

[B253] Yasumatsu K. Nagao J. I. Arita-Morioka K. I. Narita Y. Tasaki S. Toyoda K. . (2020). Bacterial-induced maternal interleukin-17A pathway promotes autistic-like behaviors in mouse offspring. Exp. Anim. 69, 250–260. doi: 10.1538/expanim.19-0156 32009087 PMC7220715

[B254] Yu D. Jain S. Wangzhou A. Zhu B. Shao W. Coley-O'Rourke E. J. . (2025). Microglia regulate GABAergic neurogenesis in prenatal human brain through IGF1. Nature 646, 676–686. doi: 10.1038/s41586-025-09362-8 40770097 PMC12527950

[B255] Yu Z. Gutu A. Kim N. O'Shea E. K. (2025). Activity-dependent synapse elimination requires caspase-3 activation. Elife 13:1779. doi: 10.7554/eLife.101779.3 PMC12151539 40492499

[B256] Zhan Y. Paolicelli R. C. Sforazzini F. Weinhard L. Bolasco G. Pagani F. . (2014). Deficient neuron-microglia signaling results in impaired functional brain connectivity and social behavior. Nat. Neurosci. 17, 400–406. doi: 10.1038/nn.3641 24487234

[B257] Zhang F. Yang W. Sun R. Duan X. Yang Y. Wang A. . (2026). Cytokine-microglia interactions in neuroinflammation: mechanisms, disease dynamics, and therapeutic strategies. Antioxid. Redox Signal. 44, 311–331. doi: 10.1177/15230864251401591 41449192

[B258] Zhang Q. Li Y. Zhang J. Cui Y. Sun S. Chen W. . (2025). IL-17A is a key regulator of neuroinflammation and neurodevelopment in cognitive impairment induced by sevoflurane. Free Radic. Biol. Med. 227, 12–26. doi: 10.1016/j.freeradbiomed.2024.11.039 39581388

[B259] Zhang Y. Watson B. Rattan A. Lee T. Chawla S. Geistlinger L. . (2025). A spatial atlas of the complement system uncovers unique expression patterns in postnatal brain development in mice. Nat. Commun. 16:11132. doi: 10.1038/s41467-025-66048-5 41331253 PMC12705658

